# Deep attention super-resolution of brain magnetic resonance images acquired under clinical protocols

**DOI:** 10.3389/fncom.2022.887633

**Published:** 2022-08-25

**Authors:** Bryan M. Li, Leonardo V. Castorina, Maria del C. Valdés Hernández, Una Clancy, Stewart J. Wiseman, Eleni Sakka, Amos J. Storkey, Daniela Jaime Garcia, Yajun Cheng, Fergus Doubal, Michael T. Thrippleton, Michael Stringer, Joanna M. Wardlaw

**Affiliations:** ^1^School of Informatics, University of Edinburgh, Edinburgh, United Kingdom; ^2^Centre for Clinical Brain Sciences, University of Edinburgh, Edinburgh, United Kingdom; ^3^Stroke Clinic, National Health Service Lothian, Edinburgh, United Kingdom

**Keywords:** super-resolution, Magnetic Resonance Imaging, deep learning, image reconstruction, explainable artificial intelligence, brain imaging, U-Net, generative adversarial networks

## Abstract

Vast quantities of Magnetic Resonance Images (MRI) are routinely acquired in clinical practice but, to speed up acquisition, these scans are typically of a quality that is sufficient for clinical diagnosis but sub-optimal for large-scale precision medicine, computational diagnostics, and large-scale neuroimaging collaborative research. Here, we present a critic-guided framework to upsample low-resolution (often 2D) MRI full scans to help overcome these limitations. We incorporate feature-importance and self-attention methods into our model to improve the interpretability of this study. We evaluate our framework on paired low- and high-resolution brain MRI structural full scans (i.e., T1-, T2-weighted, and FLAIR sequences are simultaneously input) obtained in clinical and research settings from scanners manufactured by Siemens, Phillips, and GE. We show that the upsampled MRIs are qualitatively faithful to the ground-truth high-quality scans (PSNR = 35.39; MAE = 3.78E−3; NMSE = 4.32E−10; SSIM = 0.9852; mean normal-appearing gray/white matter ratio intensity differences ranging from 0.0363 to 0.0784 for FLAIR, from 0.0010 to 0.0138 for T1-weighted and from 0.0156 to 0.074 for T2-weighted sequences). The automatic raw segmentation of tissues and lesions using the super-resolved images has fewer false positives and higher accuracy than those obtained from interpolated images in protocols represented with more than three sets in the training sample, making our approach a strong candidate for practical application in clinical and collaborative research.

## 1. Introduction

Due to its non-invasive nature and radiation-free imaging approach, Magnetic Resonance Imaging (MRI) is commonly used in clinical diagnosis to visualize soft-tissue body structures, such as the brain. However, there is an inherent trade-off between the spatial resolution of the images and the time required for acquiring them. In clinical settings, to save time, scans are often obtained at the minimal spatial resolution that allows the visual neuroradiological assessment. It is common, e.g., to ensure high spatial resolution in only one of the 3D planes (e.g., axial/horizontal), and acquire these images spaced 5 mm or more from each other, which results in poor spatial resolution in other views (e.g., sagittal or coronal/vertical planes). Also, sequence parameters and acquisition protocols differ between patients and hospitals, mainly owed to differences in scanner manufacturers and clinical manifestations. This all yields these images often impossible to process by standardized pipelines, rendering them inadequate for automated precision medicine approaches and large-scale collaborative research. Data-driven methods to upsample scans obtained in clinical settings that can preserve relevant clinical features without introducing visual artifacts could drastically speed up the diagnosis process. Hence, there is a need to automate the scan processing including providing clinically accurate priors to support clinical decision-making, in addition to facilitating clinical research (Wardlaw et al., 2010; Crowe et al., 2017).

With the recent advances in deep learning, artificial neural networks have achieved human-level performance across a range of tasks, such as image classification, natural language translation, and protein structure predictions to name a few (He et al., 2015; Wu et al., 2016b; Jumper et al., 2021). Because they are able to self-learn and extract features from high dimensional data, artificial neural networks have shown exceptional capabilities in increasing the spatial resolution (i.e., known as super-resolution (SR)) of single images (Dong et al., 2015; Ledig et al., 2017; Zhang et al., 2018). Therefore, using artificial neural networks to increase the spatial resolution of clinically-acquired MRI holds potential and has seen rapid development in recent years (Greenspan et al., 2002; Pham et al., 2017; Chen et al., 2018a). If successful, this could be especially useful for potentially reducing acquisition time requirements or reconstructing poor-quality sequences within a scanning session. Retrospective assessment of the low quality or low resolution data, e.g., to investigate whether a pathology identified at a later point in time was already present (or not) in an earlier scan of the same patient, could also benefit from SR algorithms.

### 1.1. MRI super-resolution

Research in the MRI SR field has been helped by large-scale and publicly available datasets such as Human Connectome Project (HCP) (Glasser et al., 2016) and fastMRI (Zbontar et al., 2018). We have compiled a list of models which achieved state-of-the-art (SOTA) performance in a number of these benchmark datasets and report their results [in terms of Peak Signal-to-Noise Ratio (PSNR) and Structural Similarity Index Metric (SSIM)] in the Supplementary material labeled Li_Castorina_et_al_Supplementary_file_SOTA.pdf. Nevertheless, the vast majority of these publicly available research datasets only contain high-resolution MRI scans generated in highly expensive research environments that do not reflect clinical practice. Thus, downsampling methods (i.e., subsequently represented abbreviated as *f*_downsample_) are needed to synthesize the corresponding low-resolution scans, in order to obtain low-resolution (X) and high-resolution (Y) paired samples (Chen et al., 2018a) to train and test these algorithms. The deep learning models presented so far are trained on the generated low-resolution scans $\hat{X}$ to learn to approximate the inverse function $f_{\text{downsample}}^{-1}$, such that $g\left(\hat{X}\right)\approx f^{-1}\left(\hat{X}\right)=f^{-1}\left(f\left(Y\right)\right)≃Y$. The caveat with this approach is that the network is trained on “synthetic” low-resolution samples $\hat{X}$, meaning that the output model may not perform as well on “real-world” low-resolution scans *X*, which may contain more artifacts than the estimated $\hat{X}$.

Commonly used scaling methods for downsampling natural images such as a nearest neighbor or bicubic interpolation, may not effectively represent the difference in quality between high- and low-resolution MRI scans (Chun et al., 2019). K-space truncation has been used as an alternative for the production of low-resolution MR images (Chen et al., 2018b). These are obtained by applying the Fourier transform to the image obtained from the MRI scanner, where the intensity of each point represents the relative contribution of that point's unique spatial frequency to the final image (Block et al., 2008). K-space truncation refers to the process of removing the points with higher frequencies, thus removing finer image details. Since MRI scans are obtained in k-space, this method is relatively straightforward for downsampling, but in addition to removing scan details, it can worsen visual artifacts such as Gibbs ringing, which appear as high frequency lines around areas of high contrast (Block et al., 2008; Bernal et al., 2019, 2020, 2021). A major problem of this downsampling method is that the low-resolution scans may contain consistent artifacts which can be falsely interpreted by deep learning methods as clues about the high-resolution scans, leading to poor performance on real-world data.

### 1.2. SR using convolutional neural networks

Recently, SR methods have been implemented using deep learning techniques, facilitated by the growing capacity of computing power which allows the use of deeper architectures (Pham et al., 2017). These are better than common linear interpolation methods (Dong et al., 2015), and relatively fast to compute due to the parallelization of calculations (Li et al., 2021b). Several metrics and comparisons with conventional interpolation methods (Castorina et al., 2021) show an indisputable advantage of these techniques in the problem at hand. Convolutional layers are effective in extracting features from images and scans, and stacking multiple layers allows for a more complex representation of the extracted features (Li et al., 2021b).

The Super-Resolution Convolutional Neural Network (SRCNN) introduced by Dong et al. (2015), was one of the first works that used convolutional neural networks (CNNs) to upsample RGB images. SRCNN first interpolates the low-resolution input image using bicubic interpolation to the same dimension of the target high-resolution image. The input image is, then, passed through two convolutional layers: the first extracts feature maps, while the second introduces non-linearity, mapping these features to a higher resolution representation. A final layer combines the predictions producing the reconstructed high-resolution image. This method outperformed those considered state-of-the-art at the time, such as Bicubic Interpolation, Sparse-Coding (Yang et al., 2010), Anchored Neighborhood Regression (Timofte et al., 2013), and Kernel Ridge Regression (Kim and Kwon, 2010), in image upsampling tasks.

Dong et al. (2016) further improved the SRCNN by proposing the Fast SRCNN (FSRCNN). FSRCNN does not rely on the bicubic interpolation and, instead, uses three convolutional layers to extract the features, shrink the dimensionality to map the low-resolution features into the high-resolution feature space, and learn the non-linear mapping. Then, the dimensionalities are expanded and deconvolved into the output (i.e., reconstructed high-resolution image). This method could run 40 times faster than SRCNN while achieving similar or better results.

Building on their research, Kim et al. (2016) proposed the use of Very Deep Residual Neural Networks (VDSR), which outperformed SRCNN in both, performance, as measured by Peak Signal to Noise Ratio (PSNR), and runtime, by the use of higher learning rates coupled with residual CNN layers to speed up the training process. Kim et al. (2016) also made use of gradient clipping as a solution to the exploding gradients problem.

Shi et al. (2018) used the residual connection to enhance the SRCNN architecture with global residual learning and local residual learning, which aimed to overcome the partial loss of information that increases with the depth of neural networks. Their study outperformed popular architectures such as SRCNN, FSRCNN, and VDSR in terms of PSNR and Structural Similarity Index Measure (SSIM, Wang et al. 2004). Pham et al. (2017) applied similar techniques to MRI scans, using an architecture similar to SRCNN, but with residual skip connections between layers. They found that using PSNR as their metric, residual architectures achieved better performance compared to their non-residual counterpart, even when using a larger numbers of layers. Additionally, increasing the number of layers seemed beneficial only to residual architectures.

Although the above-mentioned convolution-based methods achieved remarkable results in image SR, they relied on conventional image metrics, such as mean-absolute error (MAE), PSNR and SSIM, to evaluate upsampled images against the target high-resolution images (Dong et al., 2015; Kim et al., 2016; Shi et al., 2018). These metrics, albeit efficient to compute and easy to optimize, are not the most clinically relevant as they evaluate the quality of the entire image space (e.g., including background non-brain regions). In addition, both upsampled and target high-resolution images are needed in order to compute the mentioned image metrics, rendering it impossible to estimate how well the upsampled image is on inference if the target high-resolution image is missing, as is the case with most MRI scans acquired in clinical practice.

### 1.3. SR using generative adversarial networks

Generative Adversarial Networks (GAN), first introduced in Goodfellow et al. (2014), are deep generative models that learn to generate compelling images which resemble the distributions of real datasets. GAN consists of two modules that “learn” back-to-back: a generator and a critic (also known as a discriminator). The generator learns to generate realistic images that mimic the true data distribution, whereas the critic learns to distinguish generated data from real data. This minimax optimization objective has seen tremendous success in data generation (Karras et al., 2017; Donahue et al., 2018; Li et al., 2020), unsupervised translation (Zhu et al., 2017; Bansal et al., 2018), and many more (Pathak et al., 2016; Wu et al., 2016a). Subsequently, such critic-guided training objective has also been adopted into the realm of single-image SR (Ledig et al., 2017; Wang et al., 2018).

In the first SRGAN, in addition to the adversarial training, Ledig et al. (2017) implemented a perceptual loss function, which unlike pixel-based metrics like MAE and PSNR, calculates loss on relevant characteristics of the image. In this scheme, the total loss is calculated as the weighted sum between the adversarial loss and the results of this perceptual loss function (i.e., the VGG loss), which is a deep CNN per-se trained on the ImageNet dataset (Johnson et al., 2016). The VGG loss is calculated as the Euclidean distance between the reference image and a feature map from a pre-trained 19-layer VGG network truncated at the convolution, after activation, and right before performing max pooling. The adversarial loss, obtained from the discriminator of the GAN applied to all training images, is calculated as the average classification accuracy of all training images (0 low-resolution, 1 high-resolution).

The SRGAN was later improved by truncating the perceptual VGG loss before the activation, which resulted in sharper edges, therefore, being termed ESRGAN (Wang et al., 2018). Subsequently, Liu et al. (2019) used a two-stage SR Generator Network based on VDSR to generate a high-resolution image from which MSE loss is calculated. The pair of real and generated high-resolution images are also fed to a discriminator network which calculates a loss by distinguishing whether or not the input image is from the true or generated distributions. The pair also undergoes a Sobel filtering before calculating the edge loss. The study proposed an Edge Enhanced Hybrid (EEH) loss function which sums together the MSE loss, the Discriminator adversarial loss, and the edge loss (Liu et al., 2019). This was proposed as an alternative to perceptual loss, which, in the context of medical images such as MRI, tends to produce smoother images with spot artifacts, as the feature extraction of the VGG model is primarily trained on natural images. Additionally, training only on MSE loss usually results in blurred edges which the EEH loss avoids due to the Sobel Edge loss (Liu et al., 2019).

A potential disadvantage of GAN methods is that, although the generated output image has a seemingly “real” spatial intensity distribution, it may not match the input image (Li et al., 2021b). To tackle this, You et al. (2019) introduced GAN-CIRCLE, a framework based on CycleGAN (Zhu et al., 2017), which consists of two GAN networks, with two generators and discriminators joined together. From an input image *x*, the forward GAN will produce an SR image ŷ which is then fed to the backward GAN to produce an estimate of the input image $\bar{x}$, thus completing the circle. This circle is used to calculate the cycle-consistent loss $L_{\text{cycle}}=∥x-\bar{x}∥$ which aims at ensuring consistency between *x* and $\bar{x}$. Additionally, other losses are taken into consideration in the overall objective function: 1) Adversarial loss from the discriminator, aimed at promoting consistent distributions in the output image; 2) Identity loss, fast to calculate, used for regularization, and to refine the output image; 3) Joint Sparsifying Transform loss, calculated from two components: one which aims at maintaining general characteristics of the image such as anatomy, and a second one which reduces artifacts; and 4) Supervision loss, which is calculated from the paired real and generated samples P(ŷ, *y*) and P($\bar{x}$, *x*). This use of multiple GANs combined to learn the mapping in unpaired data has shown promising results beyond the field of computer vision (Cohen et al., 2018; Kanazawa et al., 2018; Li et al., 2021a).

### 1.4. Our contributions

We use a dataset of paired low- and high-resolution scans of the same patients, who had an MRI head scan as part of a routine clinical examination, and, within 3 months, were re-scanned at a field strength of 3 Tesla using a research protocol, after consenting to participate on a study of sporadic small vessel disease (SVD) (Clancy et al., 2021). Thus, instead of using downsampled scans $\hat{X}$, we use true low-resolution scans *X* acquired in routine clinical settings, for our framework to approximate the quality difference between low- and high-resolution scans directly *g*(*X*)≈*Y* independent of any choice of downsampling function.

We developed an attention-based and critic-guided deep learning scheme that combines the advantages of both CNNs and GANs, and those of data fusion, to upsample clinical low-resolution whole MRI scans. Different from previous studies that propose an SR algorithm to upsample a single MRI sequence despite referring to “MRI scans,” we propose and evaluate the use of data fusion for upsampling simultaneously the three MRI structural sequences most commonly acquired in a clinical MRI scan: T1-weighted, T2-weighted, and fluid- attenuated inversion recovery (FLAIR). Our data fusion method considerably improves the quality of the upsampled full scans (i.e., of the three MRI sequences taken in the scan) both, qualitatively and quantitatively, through inter-channel pass-through of information.

We also explore the effect of 1) different image co-registration methods and 2) variations in the image acquisition parameters, in the performance of the proposed framework.

In addition, we explore the attention gates of the network to explain which details are extracted at each depth of its architecture, thus improving the explainability of the upsampling process. We also incorporated GradCAM (Selvaraju et al., 2017) into the Critic to improve the interpretability of the Critic model. Moreover, the learned activation maps can assist us in identifying potential artifacts in the upsampled images and not only upsample but also reconstruct low-quality high-resolution scans.

To evaluate the applicability of our method in clinical research and, potentially, in clinical practice, 1) each of the test scans underwent the same image processing pipeline used in our clinical research studies to assess tissues and lesions, and 2) a neurologist and an experienced scan manager, independently and blind to each other's results, visually assessed all the (full) scans involved in the test subsample, to evaluate the scope of the proposed framework and its feasibility for its further application to clinical research and practice.

## 2. Dataset

The data used in this study is part of an ongoing observational clinical study of sporadic cerebral SVD, a disease of the small brain blood vessels that, if progresses, can result in stroke and/or dementia, and is currently diagnosed by the presence of specific pathological features in brain MRI scans. The primary study that provided the data (Trial registration ISRCTN 12113543) includes a comprehensive set of assessments such as blood tests, blood pressure, retinal imaging, electrocardiograms, cognitive tests, and more to elucidate which factors determine the progression and rate of changes in the disease (Clancy et al., 2021). The present study only uses low-resolution and high-resolution paired brain MRI sets from 61 patients (mean age [std. dev.] 67.14 [9.78] years old, 19 women), each containing the structural MRI sequences T1-weighted, T2-weighted, and FLAIR. These sequences differently highlight tissues and neuroradiological features including pathology (Preston, 2006) and are acquired as part of all routine clinical MRI examinations. The only inclusion/exclusion criteria applied to select our sample was the acquisition of paired low- and high-resolution scans within a small time-frame. The median time elapsed between both scans was 52 days (ranging from 8 to 95 days).

**Figure 1:**
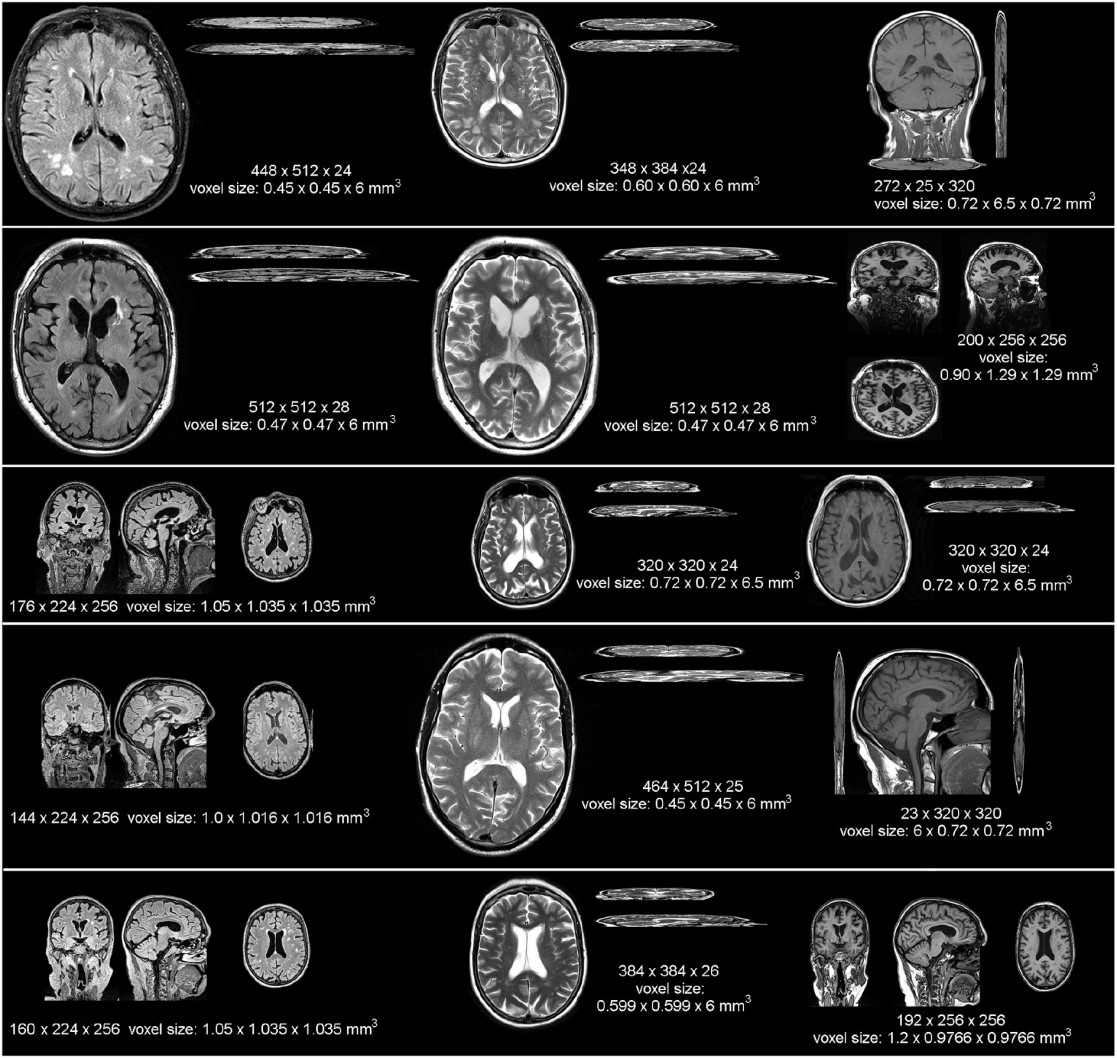
Example images from each MRI scanner and acquisition protocol (in terms of spatial resolution and sequence orientation) that provided low resolution data for our analyses, displayed at the same scale to appreciate the heterogeneity in the native resolution and in terms of image contrast and orientation (i.e., 2D, 3D, sagittal, axial, or coronal). In ascending order from top to bottom, the protocols displayed are referred in the text as protocols 1 to 5.

The low-resolution scans were obtained mainly at 1.5 Tesla (T) scanners from four different hospital sites in the Lothians and Fife regions in Scotland. Specifically, 34 were obtained from a 1.5T GE scanner model Signa-HDxt, 16 from a 1.5T Siemens scanner model Aera, 2 from a 1.5T Siemens Avanto, 1 from a 3T Siemens Prisma, 1 from a 1.5T Siemens Symphony Tim, 2 from a 3T Phillips Ingenia, 5 from a 1.5T Phillips Ingenia-Ambition X, and 1 from a 1.5T Phillips Ingenia. The sample comprises five different acquisition protocols in terms of spatial resolution and orientation of the three MRI sequences involved in the analyses. One example of a scan from each of these acquisition protocols is shown in Figure 1, referred to subsequently as protocols 1 to 5. For sequences' details, refer to Supplementary material. The high-resolution scans were all obtained at 3T using a Siemens Prisma operating a research scanning protocol, at the Edinburgh Imaging Facility, using a 32-channel head coil, which facilitates high SNR and enables higher acceleration factors. Ethical approval for the primary study that provided the image data was obtained from South East Scotland Research Ethics Committee (Ref 18/SS/0044) on 31 May 2018, and from NHS Lothian Research & Development on 31 May 2018 (Ref 2018/0084).

The validation subsample included 10/61 patient datasets. In terms of the spatial resolution and orientation of the acquisition protocol of the low-resolution scans, 1/14 patient datasets of this subsample were from protocol 1, 6/36 from protocol 2, 2/6 from protocol 3, 0/1 from protocol 4, and 1/4 from protocol 5. Also, given the time elapsed between the high- and low-resolution scans, in our evaluation we used MRI data from the first 190 patients recruited for the same clinical study, acquired in subsequent visits separated up to 3 months (i.e., the time elapsed between the low- and high-resolution scans of our main sample) with the high-resolution research protocol, as a “control” group (mean age [std. dev.] at recruitment 66.31 [11.22] years old, 59 women). Informed consent to participate in the parent study and for secondary use of anonymized data was obtained by all those who provided the images used in this study.

## 3. Methods

**Figure 2:**
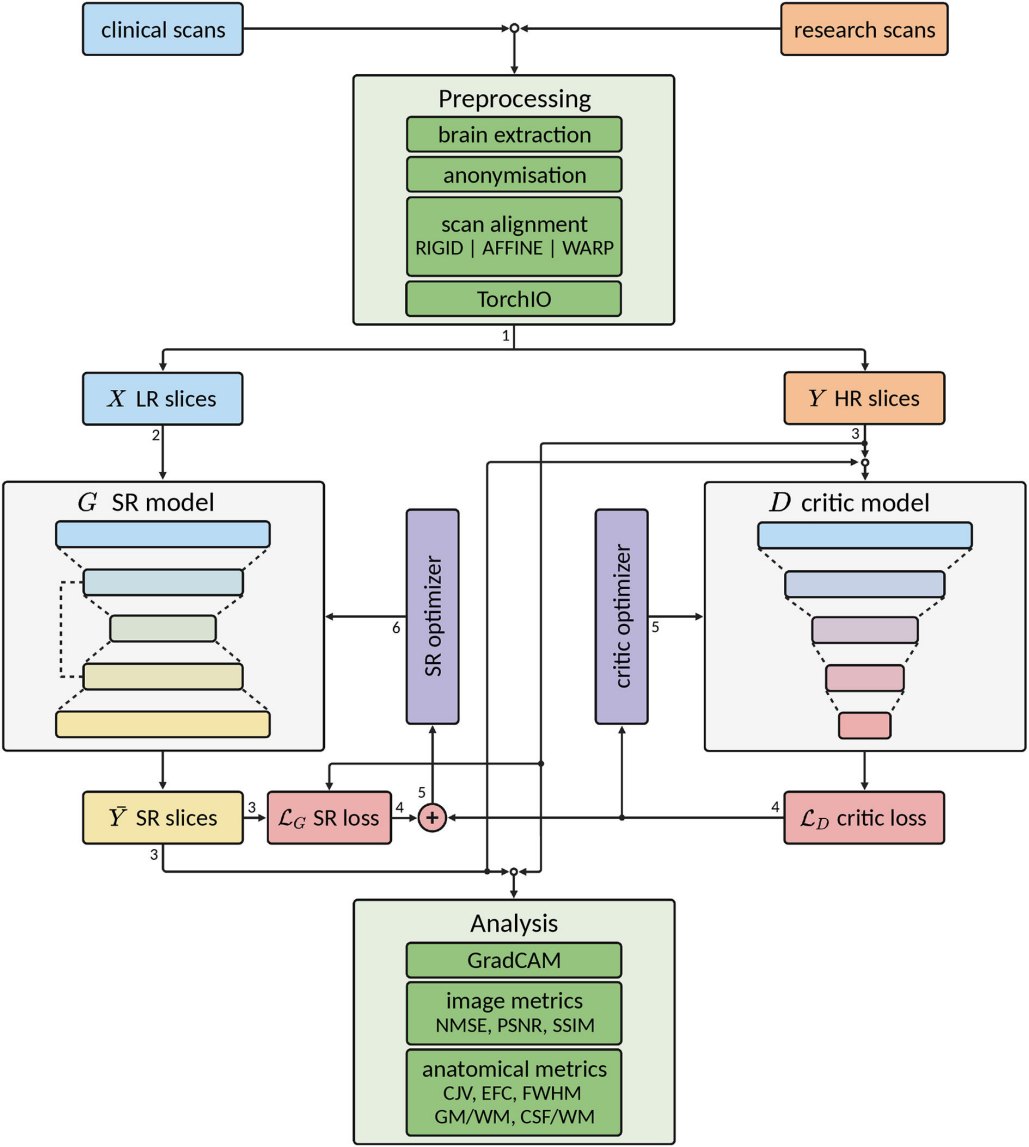
Scheme of the complete workflow of our super-resolution (SR) framework. Green colored blocks indicate pre- and post-processing operations, gray colored blocks indicate deep learning (DL) models, purple colored blocks indicate optimizers for the DL models, light blue colored blocks indicate low-resolution (LR) data, orange colored blocks indicate high-resolution (HR) data, yellow colored blocks indicate super-resolution (SR) data, and red colored blocks indicate loss calculations. Arrow lines indicate the flow of the data and the number next to them indicates the order of the data flow. Note that some operations are performed concurrently.

In this section, we detail our SR framework, including data preprocessing, generator and critic model architectures, evaluation metrics, and inference procedure. An illustration of the complete workflow is shown in Figure 2.

### 3.1. Data preprocessing

Image intensities can be influenced by instrumental and human factors, such as b1+ and b0 field inhomogeneity, the receive coil sensitivity profile, patient characteristics, and patient positioning.

Once the images are acquired and converted into NIfTI-1 format^1^, the preprocessing steps applied to all of them included correction of bias field inhomogeneities using FAST from the FMRIB Software Library (FSL, Woolrich et al., 2009) to remove the slow-varying b1 magnetic field effects that could distort the images and facilitate further brain extraction and scan alignment. The brain was extracted using the Brain Extraction Tool (BET2, Smith, 2002), also a tool from the FMRIB Software Library (Woolrich et al., 2009), to fully anonymize the data by removing all extracranial features (i.e., eyes, face, skin, and skull) from the images. All intracranial volume binary masks obtained from this step were individually visually checked for accuracy and manually edited when/if required. The brain-extracted scan sequences were then aligned to an age-/atrophy-relevant brain template (Job et al., 2017) in standard space. For evaluation, this alignment was performed, separately, in three ways:

- **Rigid**: using a rigid-body space transformation that involves only translations and rotations (i.e., 6 degrees of freedom). The shape and size (in volumetric units) of the individual brains are unaltered.
- **Affine**: using a linear transformation that, in addition to translations and rotations, also involves shearing and scaling (i.e., shrinking or enlarging, 12 degrees of freedom). This transformation, although preserves the shape by ensuring collinearity and preserving ratios of lengths of parallel planes' segments, distorts the proportions of the brain structures/features among themselves: features located toward the cortex shrink or enlarge more than features at the center of the brain.
- **Warp**: using a non-linear transformation aimed at achieving voxel-wise correspondence between the images involved, distorting not only the size but also the shape and morphology of the brain and all its features.

All space transformations were done with Niftyreg through TractoR (Clayden et al., 2011) using the default parameters defined in Modat et al. (2010). However, as all registrations involved resampling and interpolation, to evaluate their effectiveness against the results from our SR framework, linear space transformations were conducted in three ways: 1) using trilinear interpolation and the correlation ratio as cost function (default), 2) using sinc interpolation and mutual information as cost function, and 3) using spline interpolation and mutual information as cost function. As a result, images were aligned to the high resolution standard brain template, and the intracranial volume of the template was used to crop the image space to reduce sparsity. Hence, in terms of axial slices, 148 per scan (instead of 256) were processed, which represent a sample size of 27,084 (i.e., 61 full MRIs x 148 axial slices = 9,028 slices per full MRI scan; 9,028 x 3 structural sequences = 27,084 image slices). The intensity values of the MRI scan sequences were linearly rescaled (i.e., linear normalization) to the range [0, 1].

### 3.2. Network input

TorchIO (Pérez-Garćıa et al., 2021) was used to build the majority of the input pipeline. We represent each image *i* as a volume *V*_*i*_ with dimensions (*S, H, W*). In this study, each image has dimensions (148, 137, 135). We treat each volume as a 3D image of a brain slice (*S*), with height (*H*) and width (*W*). In order to upsample each slice using a 2D CNN, we expand the second dimension *C* in each image [i.e., *V*_*i*_ = (*S*, 1, *H, W*)], effectively, treating each slice as a gray-scale image. As each scan has 3 image sequences, namely FLAIR, T1-weighted, and T2-weighted, and since each sequence captures different information, we combine all 3 sequences with the hypothesis that the data fusion of the three sequences can further improve the up-sampling performance (Valdes and Inamura, 2001). To that end, we concatenate the sequences in the second dimension (i.e., (148, 3, 137, 135)) and feed the “3-channel”-images (i.e., where the 3 sequences are like “RGB channels”) to the SR model.

We evaluate two different approaches to data input to the SR model:

- Slices are randomly selected, and entire slices with dimensions (*C, H, W*) are input to the model. Zero paddings are added to the spatial dimensions such that height and width are power-of-two values.
- Patches *B* with size (*P, P*) are randomly selected from each scan, each resulting in 3D input data of dimensions (*C, P, P*). This method is commonly used in other SR works in MRI (Chen et al., 2018b; Lyu et al., 2018; Zbontar et al., 2018). Note that in this approach, depending on the number of patches *B*, not all regions of a scan may be exposed to the model.

We apply the data preparation procedure to both low-resolution and high-resolution scans in parallel to ensure the input data are in pairs. When training with mini-batching with batch size *N*, the size of the input to the model would be (*N, C, H, W*) or (*N, C, P, P*) depending on the preparation method, each requiring $\frac{S}{N}$ or $\frac{B}{N}$ steps to train one scan, respectively.

To explore the influence of training the model with FLAIR, T1-weighted, and T2-weighted sequences separately vs. doing so simultaneously, we also treat each channel individually (i.e., (*N*, 1, *H, W*) or (*N*, 1, *P, P*)). The dataset was randomly split into train, validation, and test with 40, 11, and 10 patient full MRI scans, respectively, (i.e., 5,920, 1,268, and 1,480 “3-channel-image” slices).

### 3.3. Model

**Figure 3:**
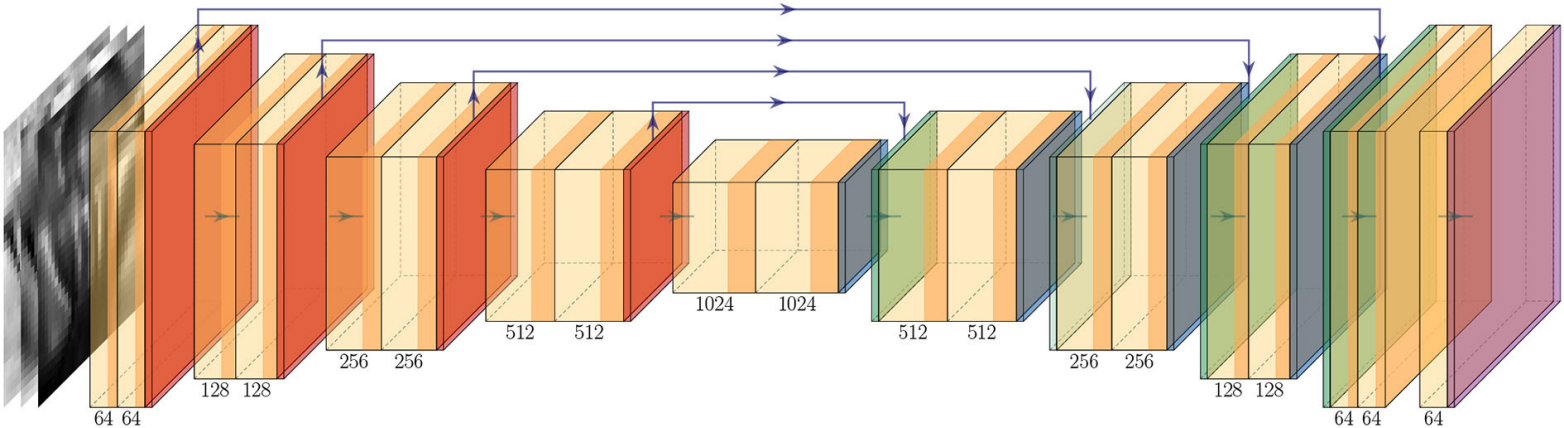
Up-sampling model *G* architecture. The two-shaded yellow block denotes a convolutional block, which consists of a convolutional layer followed by a normalization layer and an activation layer. The orange, dark-blue, green, and purple blocks denote the down-sample, up-sample, and output activation layer, respectively. The green arrow between each block represents the flow of the data and the purple arrow above represents the residual connection. The number below each block indicates the number of filters used in the corresponding block.

The super-resolution model *G* used in this study is based on the UNet architecture (Ronneberger et al., 2015), a popular model commonly used in medical image segmentation tasks, which has also shown promising results in image super-resolution study (Hu et al., 2019; Lu and Chen, 2019; Masutani et al., 2020). Briefly, the U-Net architecture can be conceptually divided into two sub-modules. 1) encoder, where data features are compressed in the spatial dimension while increasing the number of channels, usually *via* consecutive blocks of convolutional and pooling layers. 2) decoder, which mirrors the encoder, though instead of compressing, it consists of transposed convolutional layers to decompress the data in its spatial dimension while decreasing the number of channels. The output of each convolutional block in the encoder is concatenated in the channel dimension with the inputs of the corresponding convolutional block in the decoder *via* residual connections. Such architecture has shown excellent results in extracting information from input features, and the addition of residual skip connections allows very deep networks to be used while mitigating the issue of vanishing or exploding gradients (He et al., 2016). In our model, each convolutional block consists of a 2D convolutional layer with (3, 3) kernel and stride (1, 1), followed by a normalization layer and activation layer. We use Instance Normalization (Ulyanov et al., 2016) and LeakyReLU (LReLU) activation (Clevert et al., 2015). The down-sampling block consists of a max pooling layer with (2, 2) kernel, which compresses the spatial dimension by a factor of 2, followed by a convolutional block. The up-sampling block consists of a transposed convolutional layer with a stride size of (2, 2), to upsample the input variable by a factor of 2, followed by a convolutional block. When the shape of the residual connection and the input to the convolutional block differ, we apply zero padding to the variable with the smaller spatial dimension so that they can be concatenated. After the final convolutional layer, we scale the output logits to [0, 1] *via* a sigmoid layer to match the input data. Figure 3 illustrates the model architecture.

### 3.4. Model objective

To ensure that our framework can generalize on different datasets, we train the up-sampling model *G* using generic objective functions, including mean-absolute error (MAE), mean squared error (MSE), binary cross-entropy (BCE), and (maximize) the Structural Similarity Index (SSIM, Wang et al. 2004).

### 3.5. Critic

To overcome the issue of various image-based metrics not being able to capture the minute difference in MRI scans, we employ a separate critic network *D* to learn the distinction between high-resolution scan *y* and upsampled scan ŷ. The critic serves a very similar purpose to the discriminator in a GAN, with the exception that our *G* and *D* networks can be trained separately.

**Figure 4:**
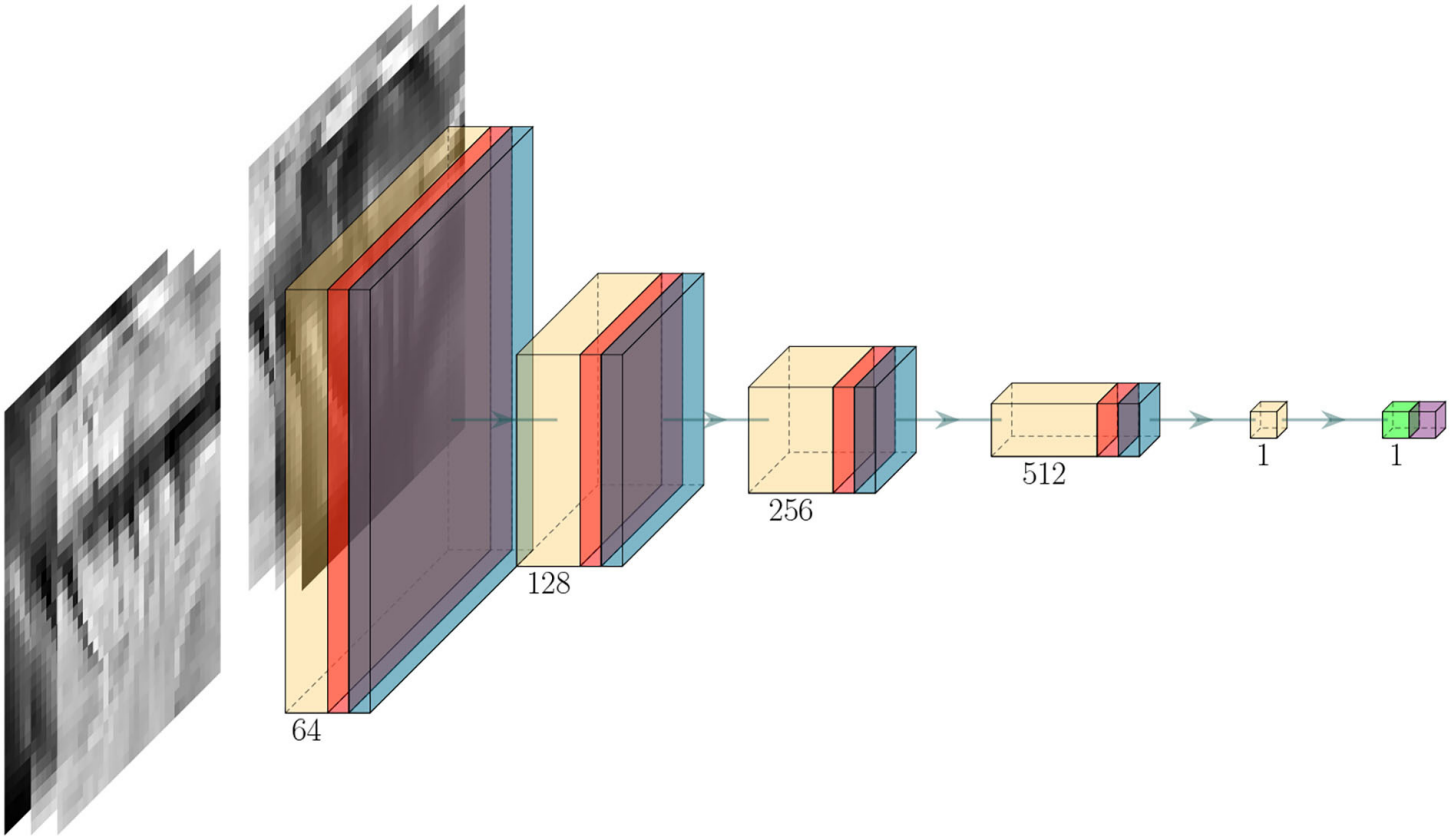
Critic architecture. Yellow, red, light blue, light green, and purple block denote convolutional, activation, dropout, dense, and output activation layer, respectively. The green arrow between each block represents the flow of data and the number below each block indicates the number of filters used in the corresponding block.

The architecture of the critic model *D* follows a similar structure as the discriminator in DCGAN (Radford et al., 2015). An illustration of the model is shown in Figure 4. The model consists of 4 consecutive blocks of convolutional layers with a kernel size of (4, 4) and stride size of (2, 2), each followed by an activation layer and dropout layer (Srivastava et al., 2014). After the final dropout layer, a convolutional layer with 1 filter is used to compress the channel information into a single value, and if the spatial dimension of the variable is larger than one, then an additional flatten layer is added to convert the 3-dimensional variable to 1-dimensional. The latent variable is then further compressed with a dense layer so that it returns a single scalar, which is then followed by sigmoid activation to output a score value.

Ideally, the critic should learn to predict a value of 1 when receiving a high-resolution input *y* and a value of 0 when receiving an upsampled input ŷ. We call this value the critic score ~[0, 1], i.e., a critic score closer to 1 indicates the input *y* resembles data from the high-resolution *Y* distribution. After each model step ŷ = *G*(*x*_low_), we feed both ŷ and *y* to the critic *D* and optimize the model with the following loss function:


(1) $${ℒ}_{D}=E[-\log D(y)]+E[-\log(1-D(\hat{y})]$$

To ensure the critic can distinguish real scans against upsampled scans, we train *D* for multiple steps (i.e., 5 steps) for every *G* update. However, due to the very limited data, the critic model over-fits the training set very quickly, hence a high dropout rate of 0.5 is needed.

#### 3.5.1. Critic-guided training

In addition, to use the critic score as a metric for evaluation, we can also use the critic model to train the SR model in an adversarial process. To that end, we can present the overall objective for the SR model *G* as:


(2) $${ℒ}_{\;G}^{\;\text{total}}={ℒ}_{\;G}+\lambda_{\text{critic}}(1-E[D(\hat{y})])$$

where λ_critic_ is the [0, 1] critic intensity hyper-parameter which allows us to control the influence of the critic on the overall optimization objective. In other words, the SR model is not solely generating a sample ŷ that closely matches its corresponding high-resolution target *y*, but samples Ŷ that should also resemble the overall data distribution of *Y*. Such formulation is very similar to the adversarial training procedure in GANs. The choice of the models (both G and D) hyper-parameters were based on the original recommendations from both Ronneberger et al. (2015) and Oktay et al. (2018), and a random search optimization procedure to find the best hyper-parameters in the validation set. Supplementary Table S2 reflects the final configurations used for the SR model G and critic model D.

### 3.6. Inference

The trained model can be used to upsample new data if the model is trained on slices (refer to Section 3.2), as each slice of the MRI scan is passed to the model. However, we also randomly generated several patches for each scan to facilitate the training process with smaller GPU memory as has been proposed previously (Johnson et al., 2016; Chen et al., 2018b; Lyu et al., 2018).

When patches were generated, each patch of a slice was upsampled. For reconstructing the output image, the upsampled patches were stitched together in a process that is handled by TorchIO (Pérez-Garćıa et al., 2021). The upsampled scans were then saved as a .mat file for further conversion to NIfTI-1 formatted files for clinical assessment by a medical professional.

### 3.7. Attention and activation maps

We incorporated some state-of-the-art methods to increase the explainability of our deep learning models. We added shortcut connections in the vanilla UNet (Ronneberger et al., 2015), to connect the output of each down-sampling block to the input of their corresponding up-sampling block *via* concatenation (e.g., purple arrows in Figure 3). In our framework, we incorporated the Attention Gate (AG) module, introduced in Oktay et al. (2018), into our up-sampling model *G*. The AG module has two inputs: the output from the previous up-sampling block, and the shortcut connection from its corresponding down-sampling block. It learns a sigmoid mask (i.e., the “attention” mask) *via* two separate convolution layers applied to the inputs. Then, the sigmoid mask is applied to the original shortcut connection. Conceptually, the AG module encourages the overall model to focus on regions of interest in the input (i.e., the shortcut connections which are a latent representation of the original input) and mask out the irrelevant information. This architecture is simple to implement and can be extended to existing UNet models with minimal modification (i.e., the only change is the variable to concatenate with). In addition to increasing the capacity of the model, the learned sigmoid “attention” masks can then be overlaid on top of the input image as a heatmap, thus allowing us to visualize the areas of interest learned by the model and verify if they are indeed reasonable. Since there are 4 down-sampling and up-sampling block pairs, we, therefore, employ 4 AG modules, each with a dimension of 32, 64, 128, and 256.

In addition to the AG modules, we analyzed the activation map of the critic model using GradCAM (Selvaraju et al., 2017). Similarly, the activation map generated by GradCAM shows areas of relevance (or irrelevance) to the final predictions; though, with one major distinction, the activation maps are not directly learned by the critic model.

### 3.8. Evaluation of the results

#### 3.8.1. Image quality metrics

After reviewing the MRI SR literature from 2017 until May 2021 and compiling the list of metrics used by each publication (refer to full list in Castorina et al., 2021), we selected four of the metrics most frequently used to evaluate image quality: MAE, Normalized Mean Squared Error (NMSE), PSNR and SSIM, to evaluate the similarity between the upsampled and the “ground-truth” high-quality scan of our testing subsample, and, at the same time, allow comparability between our results and those already published, developed for the same purpose. Briefly, MAE is the absolute voxel-wise difference, averaged over the entire scan. NMSE is the ordinary MSE normalized by the value of the ground-truth voxel:


(3) $$\begin{array}{r} \text{NSE}\left(y,ŷ\right)=\frac{{\left(ŷ-y\right)}^{2}}{y^{2}} \end{array}$$

where *y* and ŷ are voxels at the same position of the ground-truth and upsampled scans, respectively. We compute the mean across all voxels from all image slices to obtain the NMSE. Similarly, PSNR captures the relation between the maximum value of a voxel and the MSE, and is given by


(4) $$\begin{array}{r} \text{PSNR}=10\log_{10}\frac{I_{\text{max}}^{2}}{\text{MSE}} \end{array}$$

where *I*_max_ is the maximum value a voxel can take, and MSE is the unnormalized MSE. Absolute error metrics such as MAE and PSNR only compute the difference between two corresponding pixels and ignore the spatial information in the image. For example, brightening a black-and-white image can lead to high NMSE and PSNR values, yet the resulting image would be structurally identical to the original image and we would expect the error value to be small. To that end, Wang et al. (2004) proposed the Structural Similarity Index (SSIM), which considers the structural information in the images by computing the similarity between two given images *y* and ŷ *via* the weighted average of 1) luminance, 2) contrast, and 3) structure:


(5) $$\begin{array}{r} \text{SSIM}\left(y,ŷ\right)=\frac{\left(2\mu_{y}\mu_{ŷ}+c_{1}\right)\left(2\sigma_{yŷ}+c_{2}\right)}{\left(\mu_{y}^{2}+\mu_{ŷ}^{2}+c_{1}\right)\left(\sigma_{y}^{2}\sigma_{ŷ}^{2}+c_{2}\right)} \end{array}$$

where μ_*y*_ and μ_ŷ_ are the average of *y* and ŷ; σ_*y*_ and σ_ŷ_ are the variances of *y* and ŷ; σ_*yŷ*_ is the covariance of *y* and ŷ; *c*_1_ and *c*_2_ are the stabilizing terms for the denominator. The SSIM has since been used extensively in the field of computer vision and medical imaging (Dong et al., 2015; Isola et al., 2017; Ledig et al., 2017; Zbontar et al., 2018; Defazio, 2019; Zhang et al., 2019).

While these metrics are useful in quantitatively assessing the overall quality of the upsampled images, they do not clearly reflect clinically meaningful results. We, therefore, in addition, computed the following metrics:

- **Coefficient of Joint Gray and White Matter Variation (CJV)**: Quantifies the intensity variability in white matter (WM) and gray matter (GM) accounting for the overlap between their distributions (Ganzetti et al., 2016). It is calculated as:

(6) $$\begin{array}{r} \text{CJV}=\frac{\text{stdev}\left(WM\right)+\text{stdev}\left(GM\right)}{\text{mean}\left(WM\right)-\text{mean}\left(GM\right)} \end{array}$$

- **Entropy Focus Criterion (EFC)**: Uses the Shannon entropy of voxel intensities as an indication of ghosting and blurring induced by head motion (Atkinson et al., 1997).

(7) $$\begin{array}{r} \text{EFC}=\overset{N}{\underset{n=1}{\sum }}\left(\frac{B_{\text{voxel}}}{B_{\text{max}}}*\ln\frac{B_{\text{voxel}}}{B_{\text{max}}}\right) \end{array}$$

where *n* is the number of voxels and *B* is the voxel's brightness.

- **Full-Width Half Maximum (FWHM)**: The full-width half maximum of the spatial distribution of the image intensity values in voxel units. Represents the smoothness of voxels.

(8) $$\begin{array}{r} \text{FWHM}=\frac{\text{max}\left(I\right)}{2} \end{array}$$

where *I* is the intensity across all voxels.

- **White-matter to maximum intensity ratio (WM2max)**: Median intensity within the WM mask over the 95% percentile of the full intensity distribution (i.e., calculated as the 95% confidence interval of the image intensities), with values around the interval [0.6, 0.8].
- **The ratio between the summary stats of gray matter (GM), white matter (WM), and cerebrospinal fluid (CSF)**: These are mean, SD, 5% percentile, and 95% percentile of the intensity distribution in CSF, GM, and WM. For example:

$$\begin{array}{l} \frac{\text{mean}\left(I_{WM}\right)}{\text{mean}\left(I_{GM}\right)},\frac{\text{mean}\left(I_{CSF}\right)}{\text{mean}\left(I_{WM+GM}\right)},\frac{\text{stdev}\left(I_{WM}\right)}{\text{stdev}\left(I_{GM}\right)},\cdots \end{array}$$

To ensure fair comparability, the GM, WM, and CSF binary masks used in these evaluations were generated out from the brain-extracted T1-weighted images of the high-resolution subset using FSL-FAST (Zhang et al., 2001): a tool from the FMRIB Software Library.

#### 3.8.2. Comparison with state-of-the-art methods

As discussed in Section 1.1, numerous benchmark datasets were made publicly available in recent years to provide a systemic evaluation of different SR approaches (refer to Supplementary material Li_Castorina_et_al_Supplementary_file_SOTA.pdf). But the majority of these datasets contain only high-resolution MRI, requiring artificial transformations to create low- and high-resolution pairs of images, such as k-space truncation or Gaussian blurring followed by multidimensional space shrinking, unlikely to capture the properties of low-resolution MRI scans obtained from clinical settings. To that end, we compared our method against two state-of-the-art SR models, previously evaluated using the fastMRI dataset (Zbontar et al., 2018), but using our dataset instead. We implemented the vanilla UNet (Ronneberger et al., 2015) (refer to Section 3.3) and a Vision Transformer-based (ViT, Dosovitskiy et al. 2020) model as proposed by Lin and Heckel (2022), and trained them on our dataset from scratch using their default hyper-parameters.

#### 3.8.3. Applicability to clinical research-structural image segmentation

In our testing sample, we automatically segmented lesions, normal tissues, and anatomical structures in 1) images upsampled using our framework, 2) images upsampled from different types of interpolations (refer to Section 3.1), and 3) high-resolution images. We also segmented the same features in the images of the larger “control” sample, fully automatically, individually at each time point following the same procedure, to explore the variability in the longitudinal measurements using only high-resolution images acquired within the same time frame elapsed between the acquisition of the low- and high-resolution paired scans. CSF, veins and sinuses, pial layers, and normal brain tissue were segmented using Gaussian clustering of a multidimensional array formed by combining the three MRI structural sequences (i.e., FLAIR, T2-weighted, and T1-weighted images). The multispectral clustering results were subsequently optimized using the expectation-maximization algorithm. CSF-filled spaces are identified as hypointense in T1-weighted and FLAIR and hyperintense in T2-weighted. Venous sinuses and main venous pathways are hypointense in T2-weighted and with mid-to-low range intensities in T1-weighted and FLAIR. Pial layers, CSF-brain parenchyma partial volume effects and interstitial fluids, indistinguishable from each other and, therefore, classed together, are hypointense in T1-weighted and hyperintense in FLAIR and T2-weighted. Mid-range intensities in the three sequences correspond to normal-appearing gray and white matter tissues, each with characteristic image contrast depending on the sequence.

Binary masks of ischaemic lesions (i.e., these including white matter hyperintensities (WMH) and stroke lesions) were generated by thresholding the intensity values of the brain-extracted FLAIR, with a threshold equal to 1.3 × SD above the mean, using an adjusted method from Zhan et al. (2015). The resulting hyperintense areas unlikely to reflect pathology were removed automatically using a lesion distribution template generated from the segmentation results of a large study of cognitive aging (Wardlaw et al., 2011). Further refinement of the lesion segmentation was achieved by applying a Gaussian smoothing, followed by the removal of voxels with intensity values below 0.1 and with z-scores below 0.95. The WMH binary mask obtained from the high-resolution image was visually checked and manually edited to correct for inaccuracies and inclusion of the stroke lesion, for evaluating the raw results from the different upsampling methods, against the edited mask. The manual editing was performed using MRIcron^2^.

#### 3.8.4. Visual assessment of image quality

A neurologist and an experienced scan manager, independently and blind to each other's results, rated the quality of the scans of our test subsample: low-resolution, high-resolution as well as the upsampled images from the models that provided the best results using our SR framework, on a quality scale ranging from 0 to 5, being 5 high quality. Each rater was asked to outline the own criteria followed for rating the scans.

### 3.9. Code implementation and availability

The codebase^3^ was predominantly written in Python 3.8, where the core part of the deep neural networks and model training procedures were implemented using the PyTorch (Paszke et al., 2019) library and the scikit-image (Van der Walt et al., 2014) library was used for image manipulation. We experimented with the model training and inferencing on various GPUs, including the NVIDIA RTX 2060, NVIDIA RTX 2080 Ti, and NVIDIA V100, to ensure our model works with different levels of computing hardware. To further improve the model training performance, we have incorporated mixed-precision training where the majority of the computation was done in half-precision (Micikevicius et al., 2017), effectively allowing us to fit twice as much data onto the GPU memory. In addition to the implementation of our methods, the repository also contains clean, modular code for training, evaluating, and applying models to different datasets with extensive logging functionality with TensorBoard^4^. Under the current implementation, in inference, using a batch size of 1, it took on average 10 s to up-sample one scan using an Nvidia 2080Ti GPU or 40 s using a 20-cores Intel Xeon CPU.

## 4. Results

In this section, we first, present the results from sampling random patches of the scans (Section 4.1) and full scan slices (Section 4.2) in a framework that used a common objective function as a loss function. In Section 4.3, we present the results from using full scan slices as input but in a framework trained with an adversarial loss instead. We present the results of evaluating the influence of the scan alignment techniques (i.e., rigid vs. affine vs. warping) in Sections 4.4,4.5. We then compare our best model against two state-of-the-art models in Section 4.6. Results of the implementation of attention and activation gates are presented in Sections 4.7, 4.8, respectively. We show the results from evaluating the applicability of our framework in clinical research and its potential for being introduced in clinical practice, in Section 4.9. We performed a simple random search (Bergstra and Bengio, 2012) optimization to find a reasonable hyper-parameter configuration on the validation subset with affine transformation and using full slices. The exact configuration is available in Supplementary Table S2. All results presented below were generated by models (both *G* and *D*) trained on our dataset from scratch for 100 epochs using the Adam optimizer (Kingma and Ba, 2014) where all models converged.

### 4.1. Upsampling random patches

Due to the large size of the MRI scans, sampling patches (i.e., as opposed to sampling whole slices) may be necessary, depending on available GPU memory. We evaluated the effects that: 1) variations in the content of the input data (i.e., multi-channel vs. single-channel), and 2) variations in key hyper-parameters, had on our three quantitative performance metrics.

#### 4.1.1. Multi-channel vs. single channel input

**Figure 5:**
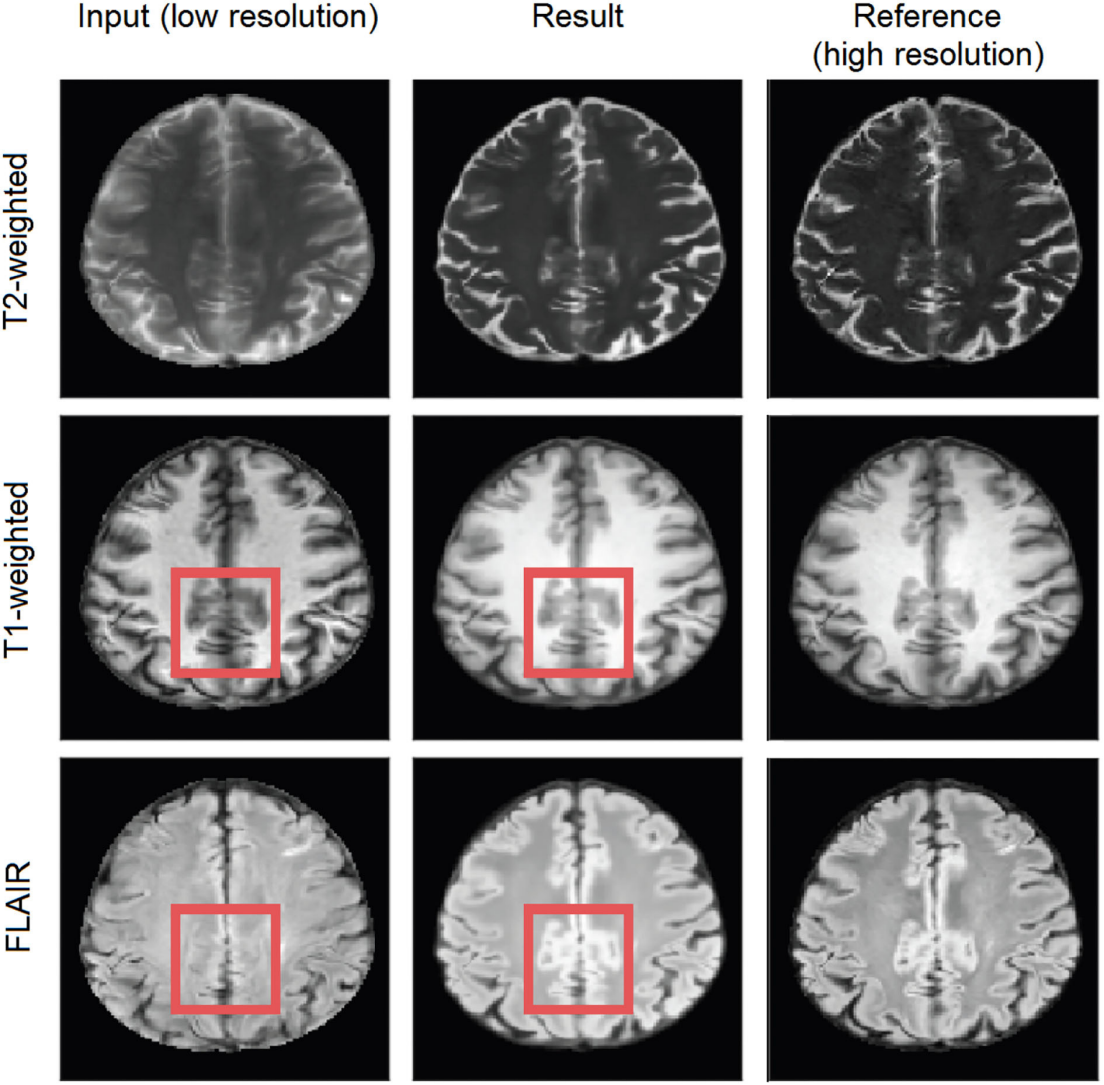
Axial slice of a scan in the test subsample. The left hand-side column shows the input low-resolution image sequences, the middle shows the generated (output), and the right hand-side column shows the target high-resolution images. The red square represents an area of possible inter-channel pass-through information.

We evaluated the effect of using a single MRI sequence as input, therefore, processing the three sequences separately, vs. using as input the three sequences (i.e., FLAIR, T1-weighted, and T2-weighted) simultaneously (i.e., multi-channel input). As Supplementary Figures S1, S4 show, the models trained with the three scan sequences simultaneously input (i.e., data fusion) yielded significantly better performance. By using multi-channel input data, information from different channels complement information that may have been missed in some areas. For instance, in Figure 5, the supramarginal gyri (highlighted in red), appear missing in the original low resolution FLAIR (i.e., due to interpolating thicker slices), but are present in the T1-weighted sequence and partially visible in T2-weighted. The upsampled scan from a model that uses the multi-channel input has the previously missing information present in all sequences, being the result qualitatively closer to the target high-resolution.

#### 4.1.2. Effect of key hyper-parameters-patch dimensions

We tested patches of dimensions: 8 × 8, 16 × 16, 32 × 32, 48 × 48, and 64 × 64. Generally, the larger the patch size, the better the performance of all the three metrics (i.e., PSNR, SSIM, and MAE). Larger patch sizes encompassed an increase in computational cost for training the model, as the batch size for larger patches is smaller and, therefore, training (and upsampling) takes longer. Refer to Supplementary Figure S1 for results.

#### 4.1.3. Effect of key hyper-parameters-number of patches

The number of patches obtained from one scan influences the MRI space explored by the network. We evaluated our framework using 100, 500, 1000, and 5000 patches, given that the volume size of each sequence is (148, 137, 135). The more patches sampled, the higher the performance. Extracting more patches come at a computational cost during training, but it generally resulted in higher upsampling (i.e., SR) performance. The best performance was obtained when using a patch dimension of 64 × 64 with the largest number of patches evaluated (i.e., 5000 patches per scan, refer to Supplementary Figures S2, S3).

#### 4.1.4. Effect of key hyper-parameters-number of filters

As shown in Supplementary Figure S4, generally, increasing the number of filters helped to improve the performance up to a certain point. Using 128 filters often yielded good performance but also showed signs of over-fitting. For our dataset, we found 64 filters to yield the best upsampling (i.e., SR) performance with the least computational requirements, offering a very good trade-off between performance, generalizability, and expressiveness.

### 4.2. Upsampling full scans slices

We evaluated using each scan's slice as input vs. patching while using as an input each sequence (i.e., FLAIR, T1-weighted, and T2-weighted) simultaneously in a multi-channel approach vs. treating each sequence separately.

#### 4.2.1. Patching vs. full slices

Super-resolution of full scan slices was possible although this required larger GPU memory. As shown in Supplementary Figure S4, using whole slices as input had a major increase in performance for single sequence input (i.e., input data split per sequence). In the models that used multi-channel input (i.e., sequences merged in a single data array), training using whole slices as input produced only a slight improvement over patching but at an increased computational cost.

### 4.3. Upsampling full scans slices with adversarial loss

The metrics commonly used for measuring the performance of SR models (Castorina et al., 2021) at times fail to consider the upsampled scan as a whole and reflect just pixel-wise differences. We, therefore, implemented the use of a critic in the training process to distinguish between generated upsampled images and real high-resolution images. In this framework design, we calculate the error rate, which is then optionally added to the loss function. As shown in Supplementary Table S1, adding the critic score to the loss function did not seem to improve the performance. However, in our proposed framework, we still train the critic in conjunction with the conventional model (i.e., that uses MAE, SSIM, and PSNR in the loss function) without adding on the loss, for being able to use GradCAM to identify potential artifacts in the upsampled scan, as discussed in Section 4.8.

### 4.4. Comparing scan alignment techniques

The numeric results obtained from using affine and rigid registration techniques for aligning the MRI sequences of the low resolution and high resolution scans to the high resolution standard brain template, can be seen in Supplementary Table S1. Warping consistently under-performed the linear registration techniques in all tests and did not render clinically useful results. From the two linear registration methods, the affine transformation gave slightly better results.

### 4.5. Best model

**Figure 6:**
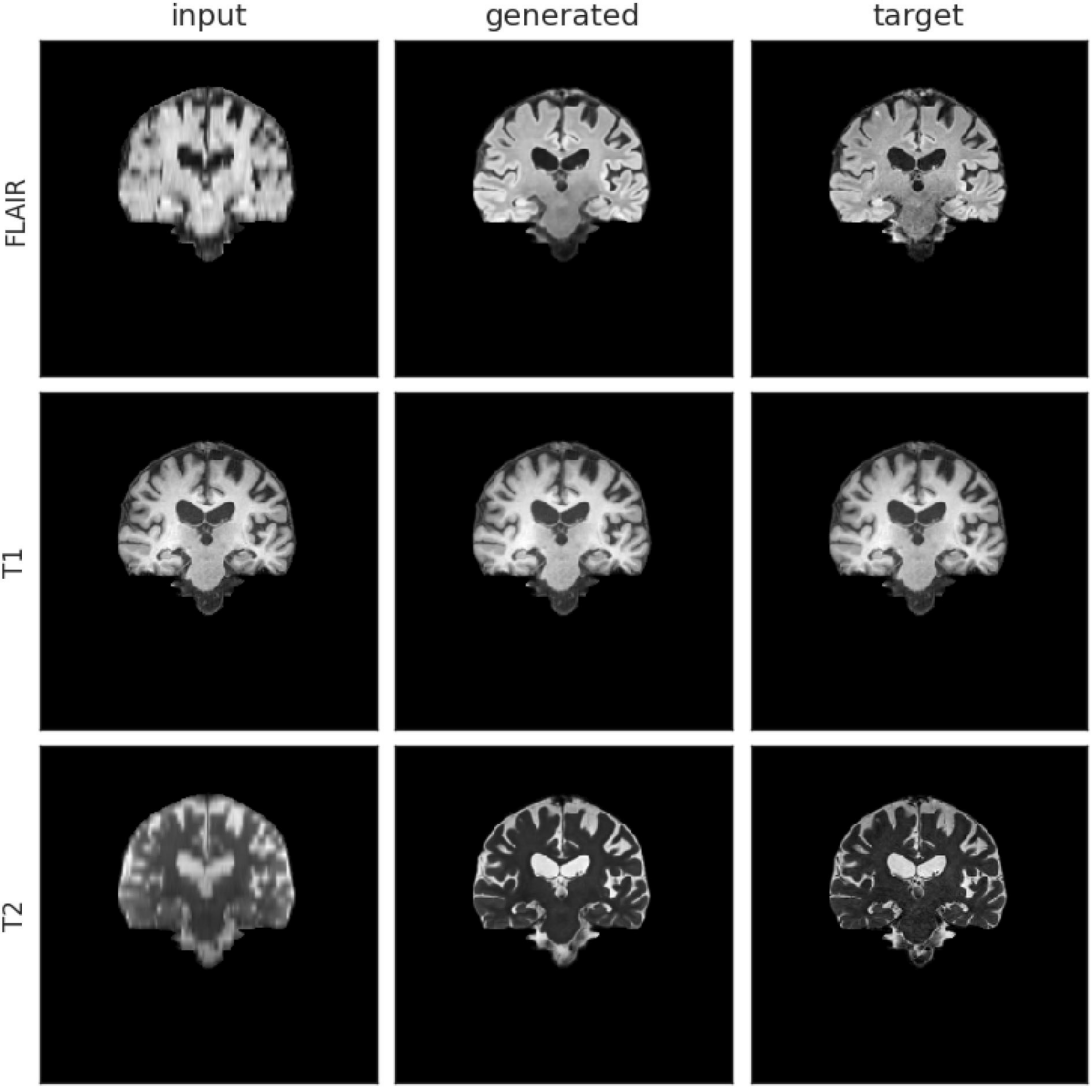
Input, generated (i.e., upsampled), and target mid-coronal slices from a scan-set with low-resolution images acquired with protocol labeled as 2. The left hand-side column shows the input images, the middle column shows the generated (output) images from Model 0 and the right hand-side column shows the target images.

Supplementary Table S1 shows the test performance of different models trained with different data pre-processing procedures and objective functions. Overall, Model 0 achieved the best result, which uses an affine transformation to align the scans to the template, and full slices of the three MRI sequences (i.e., FLAIR, T1-weighted, and T2-weighted) input simultaneously. In our validation set, we obtained MAE = 3.783E−03, NMSE = 4.32E−10, PSNR = 35.39, and SSIM = 0.9852. An upsampled scan from the low-resolution acquisition protocol 2 is available in Figure 6. The visual improvements are especially visible for FLAIR- and T2-weighted sequences, with higher contrast between white and gray matter, added details, and sharper (i.e., less blurry) images.

Of the models that used rigid-body transformation to align the input and target (i.e., reference) images to the template, Model 6 (which also used a multi-channeled full slice input approach) was the best. In the validation set, we obtained MAE = 3.88E−03, NMSE = 4.35E−10, PSNR = 35.2, and SSIM = 0.984 using this model.

**Figure 7:**
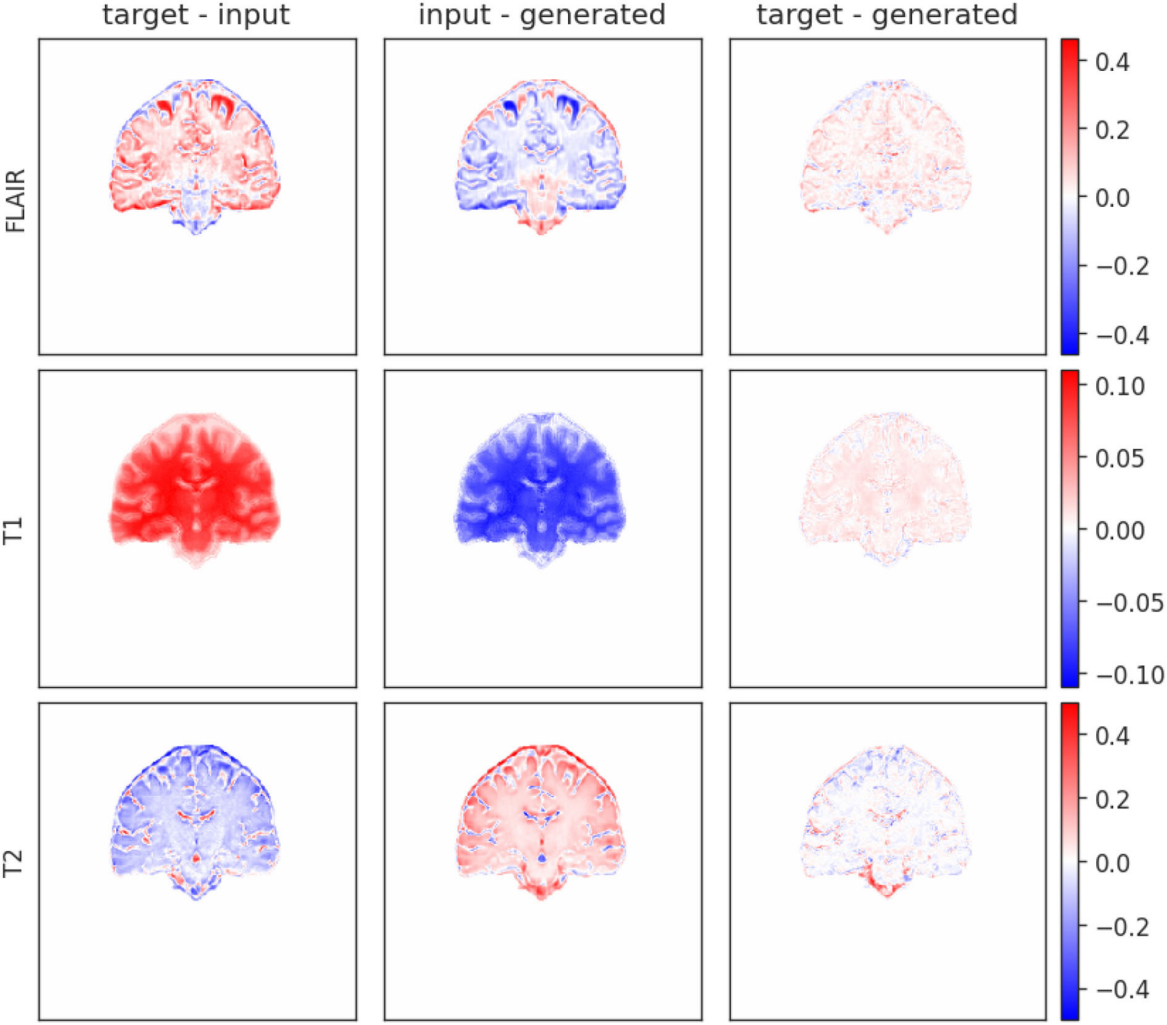
Difference map of input, target, and generated mid-coronal images upsampled using Model 0 from an MRI scan acquired with the protocol labeled as 2. The left hand-side column is the difference between target and input, the middle is between the input and the generated and the right is between target and generated.

We further compared the generated images of each model with the target high-resolution images plotting the difference maps as shown in Figure 7. As per the example shown in the figure, the difference between the brain tissue of the target and the generated SR image for the T2-weighted sequence was very low across the sample, because the tissue contrast of this sequence in the native low-resolution and high-resolution images of all the data used in testing and training was similar. Large variations in T1-weighted and FLAIR sequence acquisition parameters across the dataset used caused differences between the upsampled and target image contrasts in these two sequences.

### 4.6. Comparison with state-of-the-art methods

The mean SSIM and PSNR values (or best result as specified) of the state-of-the-art methods applied to reconstruct and/or upsample MRI, achieved on the datasets used for their development are shown in the Supplementary material^5^. Those obtained with our framework in our dataset (Supplementary Table S1), regardless of the model configuration, are comparable to the ones reported by these methods and rank among the top 10. Table 1 shows the performance of the three models from the two state-of-the-art methods implemented, trained, and tested using our dataset. While all three models yielded decent results our proposed model achieved the best overall result while having the fewest trainable parameters. Of note, the SSIM values obtained by Lin and Heckel (2022) using the Vision Transformer (ViT-M) approach to reconstruct the images from the fastMRI dataset were in the range of 0.823 to 0.926, lower than those obtained here using our dataset (i.e., 0.9443 and 0.9562).

**Table 1:** Comparison of UNet (Ronneberger et al., 2015), ViT-M (Lin and Heckel, 2022), and Model 0 (i.e., the best model, refer to Section 4.5) on our dataset with true paired low-resolution and high-resolution MRI scans.

**Method**	**Merge**	**Patch size**	**Num. Parameters**	**NMSE**	**PSNR**	**SSIM**
UNet	True	32	31M	9.63E-07	21.35	0.7398
ViT-M	False	8	32M	2.49E-09	27.68	0.9443
ViT-M	True	8	32M	1.37E-09	30.31	0.9562
ours	True	N/A	29M	**1.16E-09**	**31.08**	**0.9706**

All models were trained from scratch on our dataset with affine-registered scans, as specified in Section 3.1. We used the default hyper-parameters in UNet and ViT-M as proposed in their respective study. Merge: combine FLAIR, T1-weighted, and T2-weighted as a single input (refer to Section 3.2). The best method in each metric is in bold font for visibility.

### 4.7. Attention gates visualization

**Figure 8:**
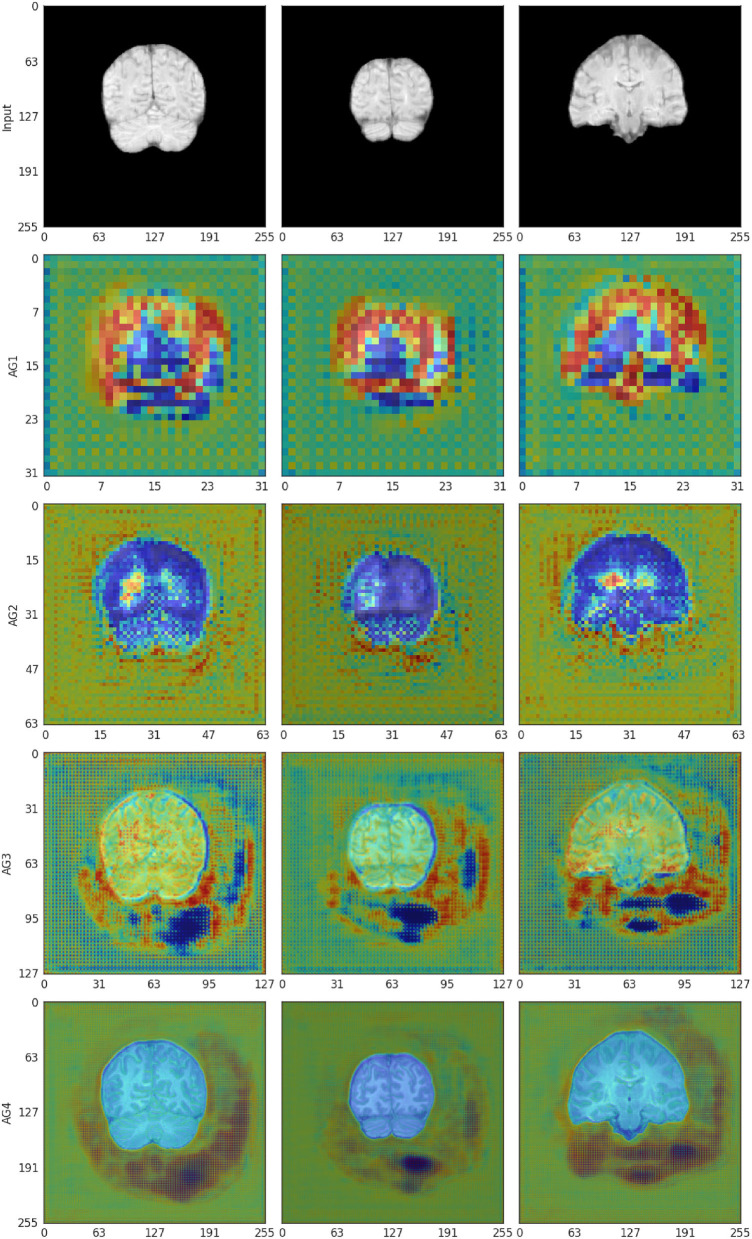
The self-learned attention masks from AG 1-4 in the self-attention Upsampler for affine-registered scans (Model 0). The attention mask is represented in a jet colormap, with areas of high attention in red and low attention in blue.

The self-learned attention maps (refer to Section 3.7) in Figure 8 illustrate the areas of focus learned by each AG module in Model 0. This makes it possible to understand which areas of the convolved image were deemed relevant by the SR model in its up-sampling process at each up-sampling stage: **AG1** (size 32 × 32) has moderate attention in the background and higher attention around the edges of the brain. Some areas of low attention are present inside the brain and at the cerebellum; **AG2** (size 64 × 64) also has a moderate attention level in the background and lower in the brain tissue, especially in the white matter. Higher attention areas are located bordering the brain and around the brain ventricles; **AG 3** (size 128 × 128) has moderate attention in both the background and the brain tissue. Brain borders and sulci have contrasting low and high attention. Background areas bordering the brain with high and low attention in AG2 appear consolidated and extended in this gate; **AG4** (size 256 × 256) compared with AG3 shows lower attention in the brain and reduced extent of the low attention areas outside. Areas of high attention are concentrated toward the inferior and lateral background surrounding the brain.

### 4.8. GradCAM activation visualization

**Figure 9:**
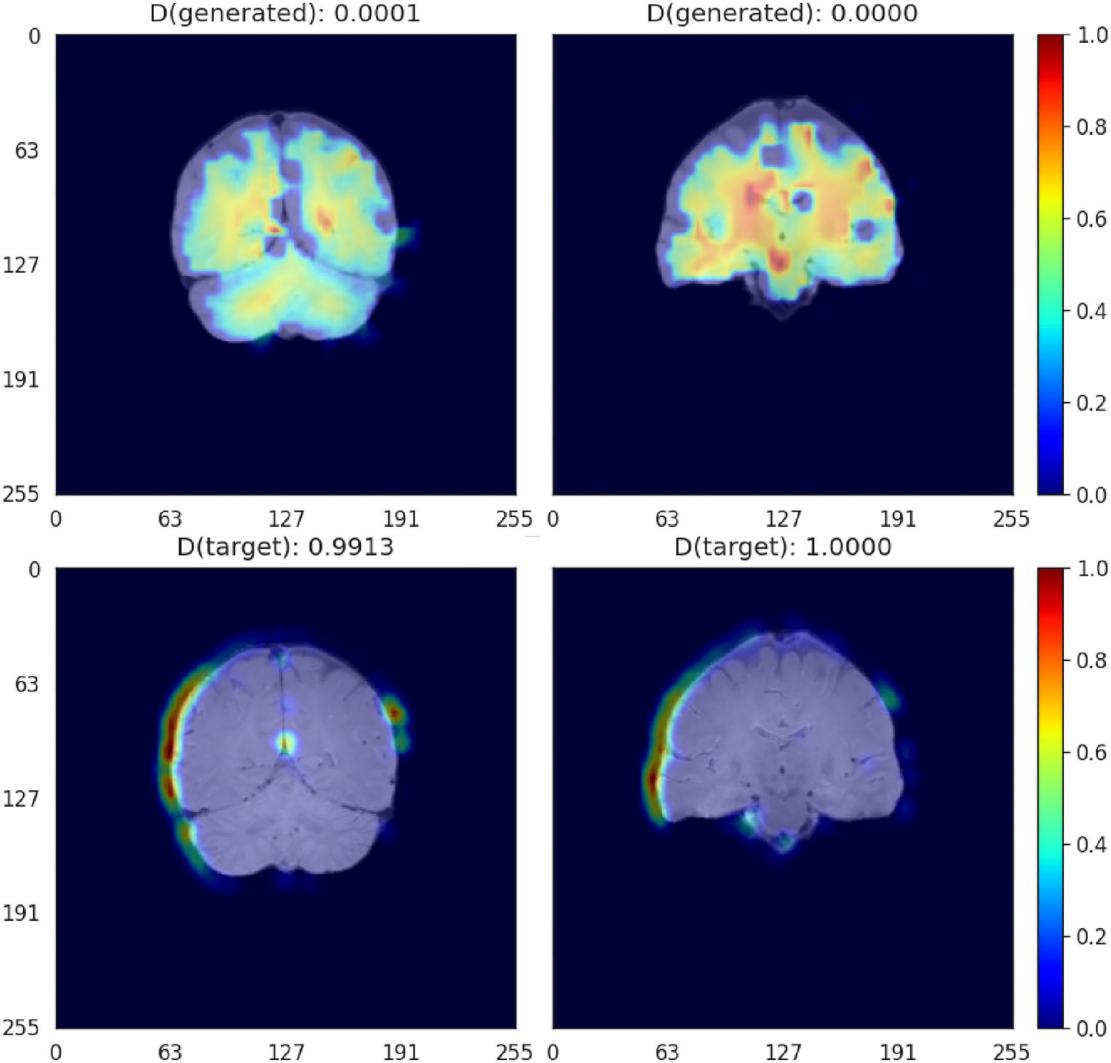
GradCAM activation plots for the Critic for affine-registered scans. Activation is represented in a JET colormap, with areas of high activation in red and low attention in blue.

Activation plots highlight areas of interest to the Critic. As shown in Figure 9, when scans are aligned to the template using affine registration (i.e., 12 degrees of freedom), in the generated images the Critic generally focuses on the white matter. In the target images (i.e., high-resolution images), the activation seems to be focused on the brain contour. The aqueduct also seems to be an area of attention in the target images (bottom left in Figure 9). When scans are aligned using rigid-body registration (i.e., 6 degrees of freedom), the pattern of activation is similar (Supplementary Figure S5).

### 4.9. Clinically relevant evaluation

#### 4.9.1. Brain image quality metrics

Table 2 shows the results of subtracting the added quality metrics calculated from the upsampled images using Model 0 and Model 6, and the low resolution images resampled to the dimensions of the high resolution images using different types of interpolations and cost functions, from the quality metrics calculated from the target high resolution images.

**Table 2:** Mean Absolute Error (MAE) and its standard deviation (STD) of the results of computing the anatomically meaningful metrics for upsampled and interpolated images against the high resolution images for each acquisition protocol in the testing subsample.

**Metric (Protocol no.)**	**Model 0**		**Model 6**		**Interpolated Spline**		**Interpolated Sinc**		**Interpolated trilinear**	
	**MAE**	**STD**	**MAE**	**STD**	**MAE**	**STD**	**MAE**	**STD**	**MAE**	**STD**
EFC (1)	18437	N/A	**9,480**	**N/A**	53,836	N/A	56,046	N/A	28,045	N/A
EFC (2)	76,065	36,119	99,743	63,486	65,339	40,743	65,720	40,366	**58,639**	**49,944**
EFC (3)	14,188	N/A	12,565	N/A	17,625	N/A	17,170	N/A	**6,318**	**N/A**
EFC (5)	55,260	N/A	45,987	N/A	34,107	N/A	33207	N/A	**10,418**	**N/A**
CJV (1)	0.03499	N/A	**0.02999**	**N/A**	0.04869	N/A	0.04874	N/A	0.09952	N/A
CJV (2)	**0.05463**	**0.05659**	0.07352	0.03924	0.08816	0.04262	0.08856	0.04239	0.07706	0.05998
CJV (3)	**0.02844**	**N/A**	0.3429	N/A	0.1595	N/A	0.1610	N/A	0.08374	N/A
CJV (5)	0.01244	N/A	**0.00580**	**N/A**	0.01444	N/A	0.01443	N/A	0.1108	N/A
GM/WM Mean Intensity Ratio (1)	0.02285	N/A	0.03899	N/A	**0.01551**	**N/A**	0.01641	N/A	0.08344	N/A
GM/WM Mean Intensity Ratio (2)	**0.03269**	**0.02544**	0.07791	0.06963	0.09623	0.07512	0.09620	0.07480	0.09952	0.06232
GM/WM Mean Intensity Ratio (3)	0.02627	N/A	0.0296	N/A	0.03597	N/A	0.03480	N/A	**0.01541**	**N/A**
GM/WM Mean Intensity Ratio (5)	0.01237	N/A	0.00882	N/A	0.00939	N/A	0.00982	N/A	**0.00600**	**N/A**
CSF/WM Mean Intensity Ratio (1)	0.1705	N/A	**0.1469**	**N/A**	0.3339	N/A	0.3319	N/A	0.2337	N/A
CSF/WM Mean Intensity Ratio (2)	**0.3133**	**0.3927**	0.4501	0.5840	0.6675	0.8260	0.6665	0.8224	0.7128	0.8975
CSF/WM Mean Intensity Ratio (3)	0.2110	N/A	**0.2104**	**N/A**	0.4060	N/A	0.4032	N/A	0.5501	N/A
CSF/WM Mean Intensity Ratio (5)	**0.1720**	**N/A**	0.1790	N/A	0.3829	N/A	0.3819	N/A	0.4487	N/A

*Model 0 was trained with affine pre-processing and BCEwhile Model 6 was trained with rigid pre-processing and BCEloss. Full details are present in Supplementary Table S1. Protocols 1 and 5 are represented in image data from only one patient (respectively). Two patient image data were acquired using protocol 3, and data from 5 patients are reflected in the results from protocol 2. N/A stands for Not Available, as STD cannot be calculated for data from only one or two sources. The best results are highlighted in bold*.

In general, with the exception of the EFC, in which the trilinear interpolation and the correlation ratio as cost function (i.e., default transformation used nowadays in clinical research) performed better for most protocols (i.e., 3 out of 4) represented in the testing subsample, in the rest of the metrics, our SR reconstructed images were closed to the target high resolution images. Better GM/WM mean intensity ratio was obtained for the images from protocol 2, which comprised more than half of the testing set, upsampled using Model 0. Individual results from the different protocols for each of the three scan sequences that are included in our models can be seen in the Supplementary material spreadsheet.

#### 4.9.2. Tissue segmentation accuracy-results in the testing set

Volumes of the WMH, CSF, veins and dural meninges, total brain and pial layers of the reference high resolution images, the SR images from Models 0 and 6, and the low resolution images resampled using different types of interpolation and cost functions, for each of the 5 test samples are given in the Supplementary Tables S3–S6. The same tables show the values of other similarity metrics that evaluate spatial agreement between the segmentation masks obtained in the high resolution sets against those in the upsampled. These are: Dice coefficient, true positive and negative fractions, and positive predicted values.

**Figure 10:**
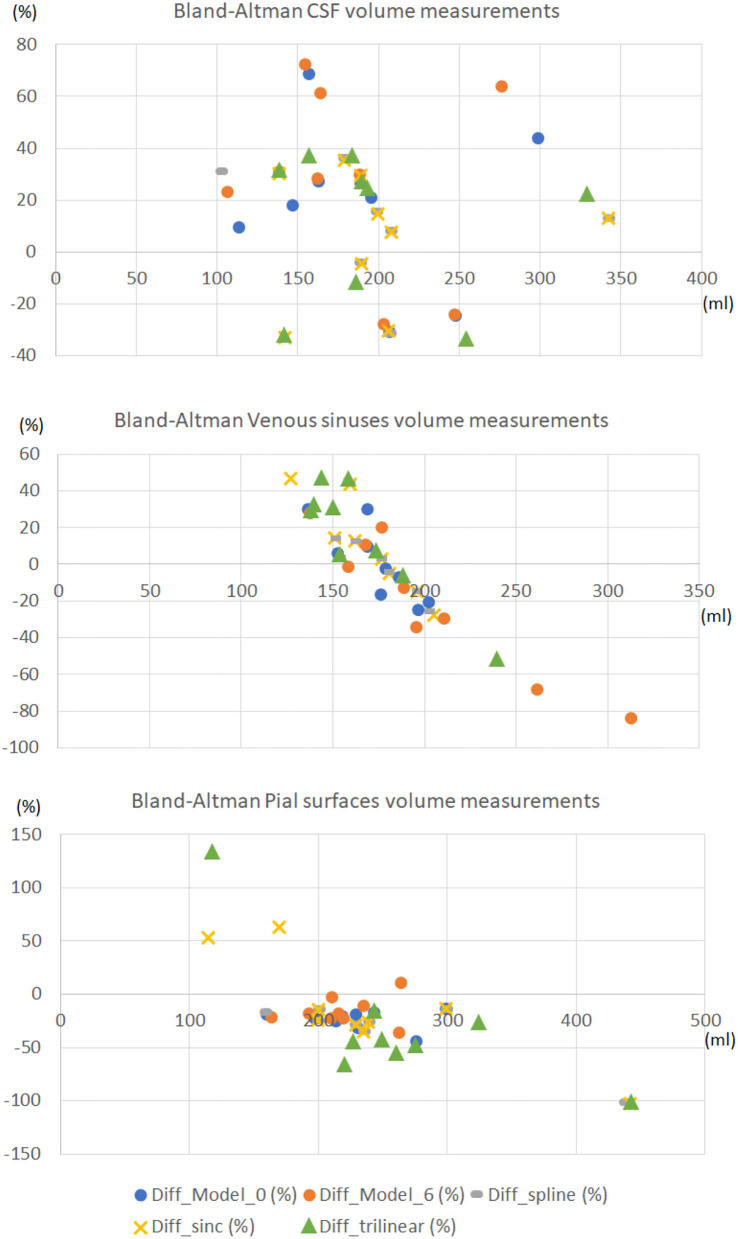
Bland-Altman plots of the agreement between the volumetric measurements obtained from the high-resolution images, and the images upsampled by different procedures, using the same scripts with identical parameters. The horizontal axes represent the average volume between the two volumetric measurements compared expressed in ml. The vertical axes represent the percentage difference between the volumetric measurement obtained from the upsampled image and the one obtained from the high-resolution (target) one, with respect to the mean value between the two measurements.

Figure 10 shows the Bland-Altman plots of the agreement between three of the volumetric measurements obtained from the high-resolution images, and the images upsampled by different procedures, using the same scripts with identical (default) parameters. With the exception of two cases with protocols under-represented in the training set, for which the SR images obtained from our framework were of lower quality, the volumetric results from our segmentations differed from those obtained from the target images in a value ranging from 0 to 20-40% of the mean value between the two measurements. This is comparable (e.g., CSF segmentation) or better (e.g., venous sinuses and pial surfaces/brain-CSF boundaries/partial volume) than the measurements obtained from the upsampled images using any type of interpolation. Specifically, the volume of the pial surfaces reported, as comprises CSF-brain parenchyma partial volume effects, is greatly affected by interpolated images as can be seen in the correspondent plot.

**Figure 11:**
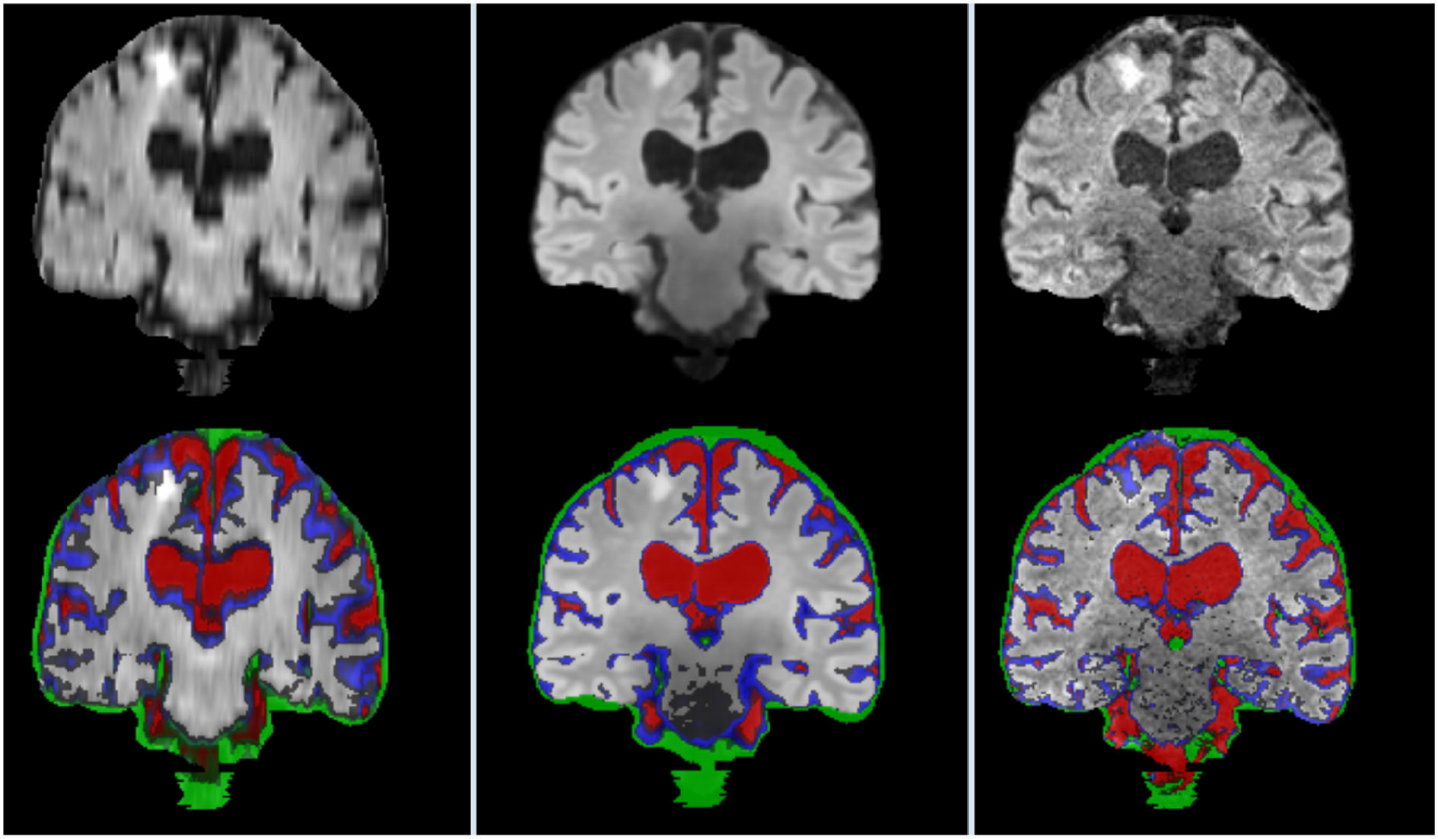
Coronal FLAIR slice of low resolution (left column) resampled and aligned to the high resolution image (right column) using trilinear interpolation, and the correspondent SR image (center column). Binary masks of CSF (red), pial layer (blue), and dural meninges and venous sinuses (green) are superimposed in the bottom row.

Figure 11 illustrates the segmentation masks of CSF-filled spaces, venous sinuses and dural meninges, and pial layers and membranes covering the brain parenchyma surface, superimposed in the FLAIR sequence, for one case, with a stroke lesion (i.e., hyperintensity) in the superior parietal cortex extending to the deep white matter visible in the coronal slice selected. The stroke is misclassified as pial structure in its entirety in the original high resolution image, and partially in the interpolated low resolution image, while correctly excluded from the masks shown in the reconstructed SR image. Normal tissues [gray (GM) and white matter (WM) masks not shown] yielded results comparable in accuracy between the high resolution and SR images, but run with different parameters and software. For example, while the FSL-FAST run with default parameters yielded consistently good results segmenting GM and WM in high resolution images, this was not the case when the SR images were used as input, and priors needed to be given for it to perform well. Still, in some cases, the SR T1-weighted images did not have enough contrast between GM and WM, and it was necessary to add anatomical knowledge to a multispectral segmentation (explained in Section 3.8.3 but considering more classes) developed in-house to obtain good results.

The results from manually editing the WMH segmentations obtained from using the high-resolution images spatially agreed better with the automatic results obtained from the upsampled scans than with those using interpolated images. Low Jaccard and Dice coefficients, in general, were in cases with prominent new and old stroke lesions manually removed from the reference segmentations, and partially included in the automated masks.

**Figure 12:**
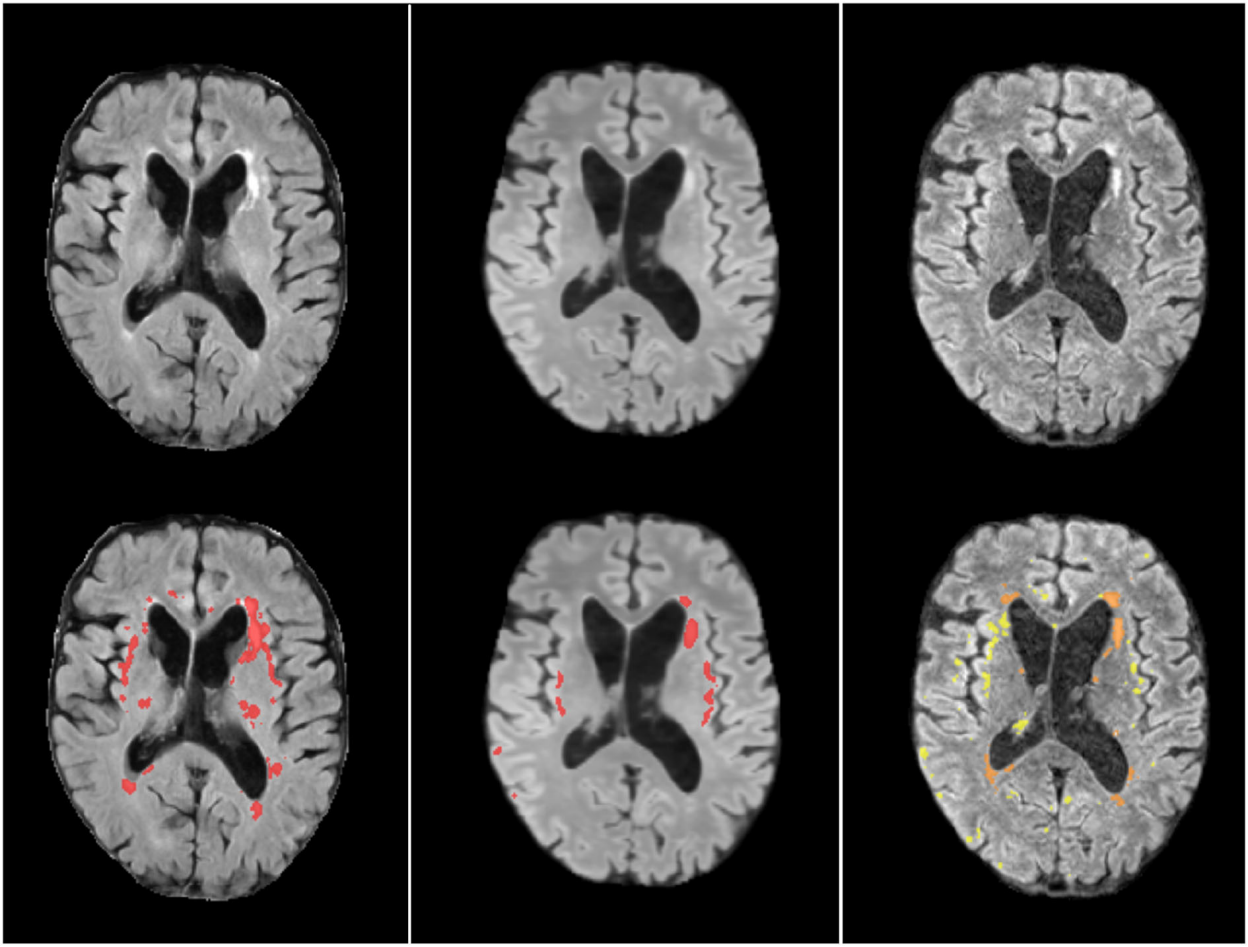
Axial FLAIR slice of low resolution (left column) resampled and aligned to the high resolution image (right column) using trilinear interpolation, and the correspondent SR image (center column). The bottom row shows the automatically generated binary masks of WMH superimposed in red for the low resolution and SR images, and in yellow for the high resolution image. The manually corrected WMH mask is also superimposed in the high resolution image and can be appreciated in an orange tone.

Figure 12 illustrates an axial FLAIR slice of a case in which the high-resolution image suffers from motion artifact and low SNR, and the low resolution FLAIR image has higher in-plane spatial resolution than the 3D-acquired images of the high resolution scan (voxel sizes of 0.47 × 0.47 × 6 mm^3^ vs. 0.973 × 0.973 × 0.973 mm^3^, respectively). As can be appreciated, the image upsampled with our SR scheme Model 0 has perceptually no motion artifacts and less noise compared to the high-resolution and less or no partial volume effect compared to the low-resolution; and the automatic WMH segmentation in the SR image has fewer false positives than the segmentation in both low- and high-resolution images. However, subtle lesions visible in the low resolution image are not reflected in the SR image, yielding few false negatives.

#### 4.9.3. Tissue segmentation accuracy-results in the control subsample

**Figure 13:**
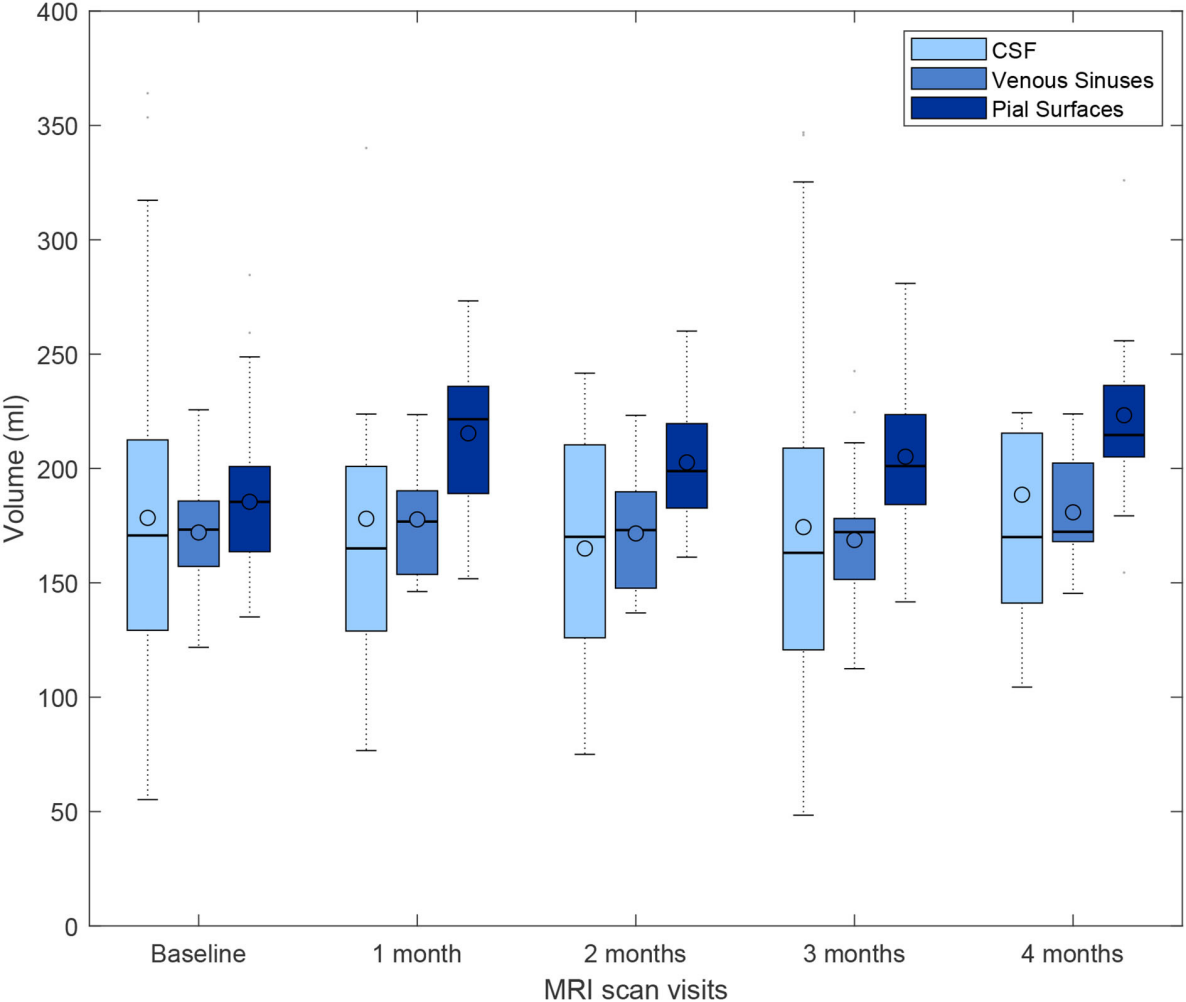
Box plots showing the full range, mean, median, and interquartile range of the volumetric measurements of CSF-filled spaces, venous sinuses, and pial layers/CFS-brain partial volume effects, for a subset of patients that underwent brain MRI scan at baseline, and subsequently, monthly up to 4 months after enrolling in the primary study that provided data for the present analysis.

From the first 190 patients recruited in the primary study that provided data for the present analysis, 188 provided MRI data at baseline, 10 at 1 month, 24 at 2 months, 64 at 3 months, and 11 at 4 months. Longitudinal MRI data in this interval, all acquired with the same high resolution research protocol, was acquired for 87 patients. We analyzed the variability in the segmentations on this subset to conclude the plausibility of the differences found between the volumetric measurements of the upsampled images and their high-resolution target images acquired within a similar interval. Figure 13 shows the distribution of the volumetric measurements of the same tissue classes for which the agreement with the upsampled results is reflected in Figure 10, not only for the baseline (reference) time point but also at 1, 2, 3, and/or 4 months. Certainly, fluctuations in the mean, median, and quartiles at each month may be due to missing values, as not all participants were imaged at the same intervals, but the subtraction of baseline volumes from those at subsequent time points showed median differences in the range of 1 to 4.72% [IQR from 0.91 to 8.21%] for CSF, 0.5 to 2.70% [IQR from 0.75 to 5.12%] for venous sinuses, and 9.24 to 12.71% [IQR from 7.60 to 14.27%] for pial surfaces/CSF-brain boundaries' partial volume.

Supplementary Figure S6 shows the distribution (and variability) of the volumes of the venous sinuses (i.e., expected to be more stable across this small period of time) in the same subsample, across the same time intervals. As can be appreciated, the differences between baseline and subsequent measurements range up to approximately ±13%, similar to the variability in the agreement between measurements obtained from the upsampled and target images in the test sample.

#### 4.9.4. Visual evaluation

Results of the visual evaluation can be appreciated in the Supplementary material labeled: Li_Castorina_Valdes-Hernandez_et_al_Visual_evaluation.xlsx. The scan manager established the following criteria for rating the images: 1) images pretty pixelated with no discernible disease markers, unsuitable for assessing them; 2) images quite fuzzy with barely visible disease markers, artifacts, most possibly unsuitable for assessing disease markers; 3) some fuzziness on images, visible disease markers such as lesions and lacunes, suitable for assessing them; 4) image quality satisfactory, very light blurriness, suitable for assessing disease markers; 5) high image quality, no blurriness, quite clear images, suitable for assessing disease markers.

As per the scan manager's evaluation, the upsampled images for protocols 2 and 3 had a superior or equal quality to the native images (i.e., native images have a low spatial resolution at least in one plane), the results from protocol 5 were poor overall, and the dataset from protocol 1 saw a slight improvement only for the T1-weighted image, but the quality of the FLAIR and T2-weighted images from the patient data acquired with this protocol was considered being slightly downgraded in the upsampling.

The neurologist graded the images according to the visibility of anatomical details and feasibility/clarity in the display of pathology. As per the neurologist's evaluation, with the exception of the images from protocol 5, for which the upsampling produced results of poor quality, T1-weighted and FLAIR were considered of equal or improved quality after being upsampled, but all T2-weighted images ranked lower than the native images. In the two cases where the target high-resolution T2-weighted images were corrupted with artifacts, the upsampled T2-weighted were considered of superior quality with respect to them, although of equal or slightly inferior quality compared to the native images.

Overall, the majority of the SR images show all anatomical details of the high resolution images with high fidelity, with the exception of the images from the patient scanned with the protocol labeled as 5, which also was underrepresented in the training sample (i.e., 4/51 datasets). Target and native images corrupted by noise saw an upgrade in the results from using the framework proposed (average score of 4/5).

## 5. Discussion

### 5.1. Upsampling patches, slices, and critic

Generally, upsampling whole slices rather than patches led to better results. This may be due to the redundancy of the patches which may not cover the full image space whilst oversampling some specific areas.

Additionally, using a critic did not improve the model's performance. We however believe that its utility goes beyond improving the accuracy, as it is a useful tool to identify potential artifacts in the upsampled images, thus increasing the explainability of the models. We propose the use of GradCAM to visualize the activations, highlighting potential artifacts, which may be useful as added information in clinical settings if the framework proposed is applied.

### 5.2. Upsampling MRI sequences separately and as channels

Using data fusion of the three MRI structural sequences (i.e., FLAIR, T1-weighted, and T2-weighted), increased the performance of our model, as information from one sequence is used in the upsampling/reconstruction of other sequences (e.g., illustrated in Figure 5). In this multi-channel architecture, the convolution process considers more than just one sequence/channel, thus allowing the flow of information between channels. As we combine FLAIR, T1- and T2-weighted much like RGB images, and input the combination as a single channel, the convolution operation is performed over the three dimensions, taking into account the three channels at once while producing the convolved image. We have not applied feature fusion, which could be explored in the future to improve the extraction of features in the different channel spaces. However, the UNet architecture with skip (i.e., residual) connections should account for features extracted early on in the convolutional processes and account for them in the later depths of the network.

### 5.3. Interpreting attention gates

We proposed the use of attention gates to visualize the attention of the model at each step of the upsampling process as shown in Figure 8. We found a peculiar pattern, where the attention of AG2 and AG4 was complementary to their respective previous gates, indicating that the network focuses on different areas of the images at different depths. It is likely that the network is learning to extract different levels of details at different gate dimensions. The lower gates might be differentiating the brain from the background while the higher gates might be focused on more specific details such as cortical folds and gray matter.

Additionally, AG2 and AG3 also cover areas of the brain which contain SVD clinical neuroradiological features, thus further research could explore the effects of hyper-parameters and their effect on the quality of upsampled areas covering the disease-specific features.

We believe that attention gate plots are essential tools in the SR field to increase the explainability of the process and results. Currently, super-resolution models are understood as black-boxes, making them potentially sensitive to the bias in the training data and, consequently reducing their usefulness in practical scenarios.

Further investigations of these maps may include channel-wise attention to differentiate attention for each sequence and understand to which extent one sequence, or an area of the sequence, is contributing toward the final upsampled image.

### 5.4. Insights from the clinically relevant evaluation of the SR framework's performance

#### 5.4.1. Datasets

The framework proposed yielded very good results for datasets acquired with protocols represented three or more times in the training set; especially in cases when the input low-resolution sequences of the scan were heterogeneously acquired, e.g., one sequence acquired axially, other coronally, and the other 3D or sagitally; or when the sequences were acquired in the same orientation but with different voxel sizes and spatial resolutions. When the images that conform to the scans are homogeneously acquired in terms of orientation and spatial resolution, the framework proposed may have limited utility, given that the information missing in the low-resolution image sequences in their native space may not be synthesized properly.

Seeking the generalizability of our approach by using a larger sample, we also pre-trained both *G* and *D* on the publicly available fastMRI (Zbontar et al., 2018) and Human Connectome Project (Van Essen et al., 2012) datasets, fine-tuning the model afterward with our data. But we did not observe a noticeable improvement in the performance of the resulting model on our validation set, hence these results are not part of the work presented. As using any image dataset acquired under research protocols for training entitles artificial downsampling, the SR scheme is likely to “reverse engineering” the operations performed rather than using the cross-channel information to add the missing information and reconstruct the input image with higher resolution. Therefore, the use of routinely acquired images in clinics is crucial for the task undertaken.

Due to the nature of the clinical condition of the patients that provided imaging data (i.e., diagnostic scan acquired soon after presenting with mild stroke symptoms), and the resources required to obtain paired scans, the dataset of paired low- and high-resolution scans available was comprised from only 60 samples (i.e., one low-resolution scan from the testing sample did not have a corresponding high-resolution scan acquired in the acceptable time frame). This limits the amount of training data for our model and, therefore, its performance. Also, all scans available were from patients with sporadic SVD, who presented to a clinic with a mild to moderate stroke symptoms. Patients with other diseases/brain anomalies (e.g., tumors, large hemorrhages, brain injuries, Alzheimer's or Huntington's diseases, rare genetic neuropathies), or in a wider age range (e.g., children, adolescents) were not represented in the data. Therefore, inaccuracies of a wider range than those discussed and presented in the Results would be expected if scans from other than patients with sporadic SVD are input. With MRI scans becoming a more common practice, we expect more training data to be available in the future for re-training our model and improving its performance. Nevertheless, given that the most represented protocols in the training dataset are those used throughout the years in the two largest hospitals in the region, and the high incidence of sporadic SVD, this framework “as is” will allow the use of a large amount of scan data for the development of robust AI-powered precision-medicine schemes.

#### 5.4.2. Representation of clinically relevant details

It is difficult to assert the extent to which the SR framework proposed reduces or enhances subtle clinically relevant details or not, given that the input and target images (used for training and testing) were acquired in a time frame up to approximately 3 months apart. As all data used were obtained from clinical settings, and resources were not available to allow both, high- and low-resolution scans, to be acquired on the same day or consecutive days, subtle lesional changes present in the low-resolution scan were sometimes absent in its SR reconstruction. As the low-resolution data was acquired soon after patients presented with stroke symptoms, subtle differences in the MRI are expected to occur in the interval elapsed between both scans. Results show that the SR framework proposed is useful in reconstructing scans that have been affected by image artifacts, which, otherwise have limited usefulness or even lack it. The proposed model could be applied, therefore, to reduce scan duration without compromising the quality of the scan when patients cannot tolerate a longer scanning time.

#### 5.4.3. Feasibility for automated precision medicine applications

While the WMH, total brain tissue, non-brain tissue structures, and CSF-filled spaces could be consistently segmented with the same parameters using SR, original, and interpolated image data, allowing comparison of the results, this was not the case for normal tissues (i.e., WM and GM). These needed separate adjustments (i.e., priors and anatomical reference locations in a multispectral approach that used the three sequences) for yielding good results using SR images, thus contrasting with the good segmentations that are obtained from using only T1-weighted images acquired under research protocols as input to FSL-FAST, which is considered the brain tissue segmentation tool for excellence. Given the small size of the training dataset, and the low (or even absent) WM/GM contrast in some of the T1-weighted images acquired in clinical settings (i.e., used for training), this is not surprising. Re-training the model with more datasets could perhaps overcome this hurdle. However, in addition, it is worth noting that, for applying our CNN-based SR framework, we linearly normalized the image intensities to double-precision values in the interval [0,1], so linearly rescaling back the intensities of the upsampled images to a full-scale 12-bit resolution integer values do not necessarily ensure fidelity in the intensity adjustment process. Future studies can evaluate the gain (if any) in applying such a more complex segmentation pipeline instead of using FSL-FAST with a single-sequence input, to the original and high-resolution scans.

### 5.5. Limitations and future study

In addition to the previously mentioned limitations related to representativeness and size of the data available, transmit radio-frequency field inhomogeneity and inhomogeneity of the receive coil sensitivity, for which compensatory methods are imperfect, can be affected by individual patient characteristics, scanner/coil properties and patient positioning, introducing a non-reproducible bias in the input data. In this study, we also expect the low- and high- resolution scans to have the same dimension. This was done by either using rigid, affine or warping space transformations of the scans to a template. In future studies, this pre-processing step can be optimized, and this optimization is integrated as part of the data processing pipeline.

The fact that the template and high-resolution images had nearly 1mm^3^ voxel dimensions and some of the low-resolution images had double the in-plane resolution of the target images (despite having only few slices), influenced the neurologist's preference for the low-resolution scan for visual diagnostic assessment in some cases. Brain template and full scans with higher spatial resolution and isotropic voxels were not available. This limitation, however, reflects the present challenges that the whole fields of medical image analysis and precision medicine need to overcome in the near future. The use of transfer- or continuous-learning techniques with higher-resolution brain templates from higher-resolution isotropic brain scans will be beneficial not only to enhance the applicability of our framework but to advance the whole field.

Another limitation is that the GradCAM activation map was averaged per channel, which is acceptable for identifying areas of interest to the model. However, for clinical use, using a channel-wise localized attention map could trace the activation onto the specific MRI sequence that provides (vs. lacks) the information the network is synthesizing at each time and location. Similarly, this applies to our attention map obtained from the attention gates, meaning our model cannot learn fine-grained attention per sequence. Future study on channel-wise attention would identify specific areas in the sequence relevant for the upsampled scan as well as further understanding the inter-channel information transfer. These will generally improve the explainability of the upsampled scans with clinically useful information.

Future study could also explore the use of VGG perceptual loss as described in Johnson et al. (2016) to enforce the performance to be reliant on the overall quality of the image rather than on pixel-wise metrics.

## 6. Conclusion

We have presented a framework for upsampling and reconstructing low resolution and low quality scans acquired in clinical practice to facilitate their integration in research settings and, therefore, the collaboration between sites. The proposed model shows promising for being applied to clinical research and precision medicine, and for adequately synthesizing information from incomplete or corrupted sequences; making it a strong candidate for being applied in clinical settings to reduce scanning time without compromising the quality of the scan. In addition, we have shown that multi-channel data fusion and input data heterogeneity are essential to achieving quality in our framework's output.

## Data availability statement

Code is available from: https://github.com/bryanlimy/clinical-super-mri. Numerical data can be found in the Supplementary Materials. Please, see imaging data availability statement at: https://github.com/bryanlimy/clinical-super-mri/tree/main/data#data-availability.

## Ethics statement

All patients gave written consent to participate in the primary study that provided data for the present analysis, and to make the data available for research purposes. The primary study that provided the data, received ethical approval by the Southeast Scotland Regional Ethics Committee (reference 18/SS/0044).

## Author contributions

BL, LC, and MV study and algorithm design and implementation, image processing and analysis, writing, editing, and revising the manuscript. ES image processing, data analysis (visual assessment) and revising the manuscript. UC and DJ patient recruitment, data acquisition, editing, and revising the manuscript. SW data acquisition, editing, and revising the manuscript. YC data analysis (visual assessment), editing, and, revising the manuscript. MV and AS study and algorithm design, project supervision, editing, and revising the manuscript. MT and MS image protocol design, image acquisition and quality control, revising the manuscript. FD and JW primary study design, editing, and revising the manuscript. All authors contributed to the article and approved the submitted version.

## Funding

BL and LC are supported by the United Kingdom Research and Innovation (grant EP/S02431X/1), UKRI Centre for Doctoral Training in Biomedical AI at the University of Edinburgh, School of Informatics. YC is supported by the China Scholarship Council. MV is funded by the Row Fogo Charitable Trust Grant no. BRO-D.FID3668413. SW is funded by the Stroke Association Post-doctoral Fellowship (SAPDF 18/ 100026). This study is also partially funded by the Selfridges Group Foundation under the Novel Biomarkers 2019 scheme (ref UB190097) administered by the Weston Brain Institute, and the Fondation Leducq Transatlantic Network of Excellence for the Study of Perivascular Spaces in Small Vessel Disease, ref no. 16 CVD 05. UC is funded by the Stroke Association Princess Margaret Research Development Fellowship 2018. FD is funded by the Stroke Association Garfield Weston Foundation Senior Clinical Lectureship(TSALECT 2015/04). DJ is Funded by the Wellcome Trust. The images used in this study were funded by the UK Dementia Research Institute which receives its funding from DRI Ltd., funded by the UK MRC, Alzheimer's Society and Alzheimer's Research UK. The 3T MRI Research scanner at the Royal Infirmary of Edinburgh, where the high-resolution images were acquired, is funded by the Wellcome Trust (104916/Z/14/Z), Dunhill Trust (R380R/1114), Edinburgh and Lothians Health Foundation (2012/17), Muir Maxwell Research Fund, Edinburgh Imaging, and The University of Edinburgh.

## Conflict of interest

The authors declare that the research was conducted in the absence of any commercial or financial relationships that could be construed as a potential conflict of interest.

## Publisher's note

All claims expressed in this article are solely those of the authors and do not necessarily represent those of their affiliated organizations, or those of the publisher, the editors and the reviewers. Any product that may be evaluated in this article, or claim that may be made by its manufacturer, is not guaranteed or endorsed by the publisher.

## Footnotes

^1^ https://nifti.nimh.nih.gov/nifti-1/

^2^ https://www.nitrc.org/projects/mricron

^3^The software codebase is attached in the submission as Supplementary material, and will be made publicly available upon acceptance.

^4^ https://www.tensorflow.org/tensorboard

^5^Li_Castorina_et_al_Supplementary_file_SOTA.pdf.
