# Supplementary material for: Deep attention super-resolution of brain magnetic resonance images acquired under clinical protocols
Source: Front Comput Neurosci. 2022 Aug 25;16:887633. doi: 10.3389/fncom.2022.887633 (PMC9458316; doi:10.3389/fncom.2022.887633)
Supplement: Supplementary file 1 [file Data_Sheet_1.pdf]

Supplementary Material

1 SUPPLEMENTARY TABLES AND FIGURES

1.1 Detailed Model Configurations and Results

Table S1 contains details of the settings used for each model evaluated as well as the performance metrics. Seed refers to the random seed used for better reproducibility, though GPU-accelerated model training can produce slightly different results depending on hardware.

Table S1. Test performance for models in different configuration

NAME	PRE-PROCESSING	λ_{CRITIC}	LOSS	SR LOSS	MAE	NMSE	PSNR	SSIM
0	AFFINE	0	BCE	0.69592	0.003783	**4.32E-10**	**35.39**	**0.9852**
1	AFFINE	0	BCE	0.69600	**0.003752**	4.46E-10	35.25	0.9847
2	AFFINE	0	BCE	0.69601	0.003762	4.44E-10	35.29	0.9846
3	AFFINE	0	MAE	0.00372	0.003779	4.52E-10	35.21	0.9845
4	AFFINE	0	BCE	0.69596	0.003901	4.76E-10	34.92	0.9845
5	AFFINE	0	BCE	0.69601	0.003831	4.69E-10	35.04	0.9843
6	RIGID	0	BCE	0.69624	0.003878	4.35E-10	35.25	0.9842
7	AFFINE	0	BCE	0.69592	0.003796	4.65E-10	35.08	0.9841
8	RIGID	0	BCE	0.69626	0.003962	4.55E-10	35.07	0.9841
9	RIGID	0	BCE	0.69630	0.004009	4.68E-10	34.96	0.9839
10	RIGID	0	BCE	0.69627	0.004001	4.68E-10	34.98	0.9838
11	RIGID	0	BCE	0.69624	0.004044	4.68E-10	34.93	0.9837
12	RIGID	0.1	BCE	0.69621	0.004028	4.70E-10	34.93	0.9832
13	AFFINE	0	BCE	0.69606	0.004049	5.23E-10	34.59	0.9828
14	RIGID	0.5	BCE	0.69626	0.004109	4.72E-10	34.88	0.9824
15	AFFINE	0	BCE	0.69604	0.005163	6.63E-10	33.54	0.9770
16	AFFINE	0	BCE	0.69604	0.005337	7.05E-10	33.26	0.9758
17	AFFINE	0	BCE	0.69604	0.005656	7.18E-10	33.11	0.9735
18	RIGID	0.5	BCE	0.69547	0.012936	3.18E-09	26.54	0.9514
19	AFFINE	0.5	BCE	0.69666	0.014573	4.28E-09	25.33	0.9197
20	WARP	0	MAE	0.01962	0.021047	3.47E-09	26.52	0.8985
21	WARP	0	BCE	0.70339	0.020610	3.36E-09	26.64	0.8970
22	WARP	0.1	BCE	0.70327	0.022140	3.82E-09	26.07	0.8798

1.2 Hyper-parameter Search Results

In Figure S1, we tested the effect of varying patch size: 8×8, 16×16, 32×32, 48×48, and 64×64 on the performance metrics `PSNR`, `SSIM` and `MAE`. We also present the same patch dimensions using each MRI sequence as a channel (merge). Generally, higher patch dimensions tend to give better results when each sequence is considered separately, while when merged, the performance is better and the patch dimension becomes less important.

In Figure S2 and S3, we test the effect of varying number of patches and patch dimension on performance metrics, merging each sequence as a channel (merge) and separately (split) respectively. Generally the higher the number of patches, the higher thee performance at every patch dimension.

Table S2. Details of the configuration of the super-resolution model G and critic model D after a random hyper-parameter search optimization on the dataset with affine transformation and without random patching.

HYPER-PARAMETER	SR MODEL G	CRITIC MODEL D
NUM. INITIAL FILTERS	32	64
ACTIVATION	LEAKYRELU	LEAKYRELU
NORMALIZATION DROPOUT	LAYERNORM	LAYERNORM
LEARNING RATE	0.25	0.5
NUM. PARAMETERS TOTAL	0.001	0.002
MEMORY USE (MB)	$\sim$7.9 MILLIONS	$\sim$394 THOUSANDS
	$\sim$850	$\sim$13.8

Finally, in Figure S4 we test the effect of varying the filter size on the performance metrics for both merge and split sequence channels, using full scan slices or patches. Generally, higher number of filters improves the performance slightly, after about 64. "Merge" and "full scan slices" is the best performing combination in terms of `PSNR`, `SSIM` and `MAE`.

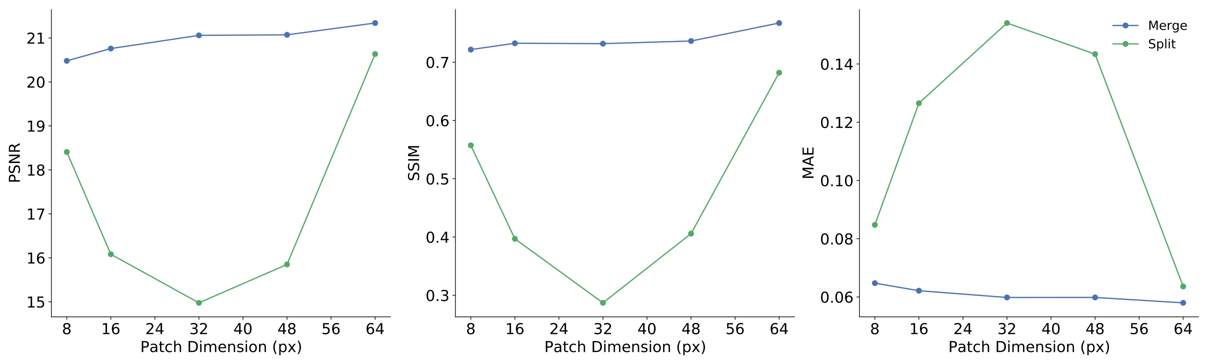

Figure S1. Performance metrics for merged (i.e. merged T1-weighted, T2-weighted, FLAIR channels) and split (T1-weighted, T2-weighted, FLAIR input separately) with increasingly larger patch dimensions

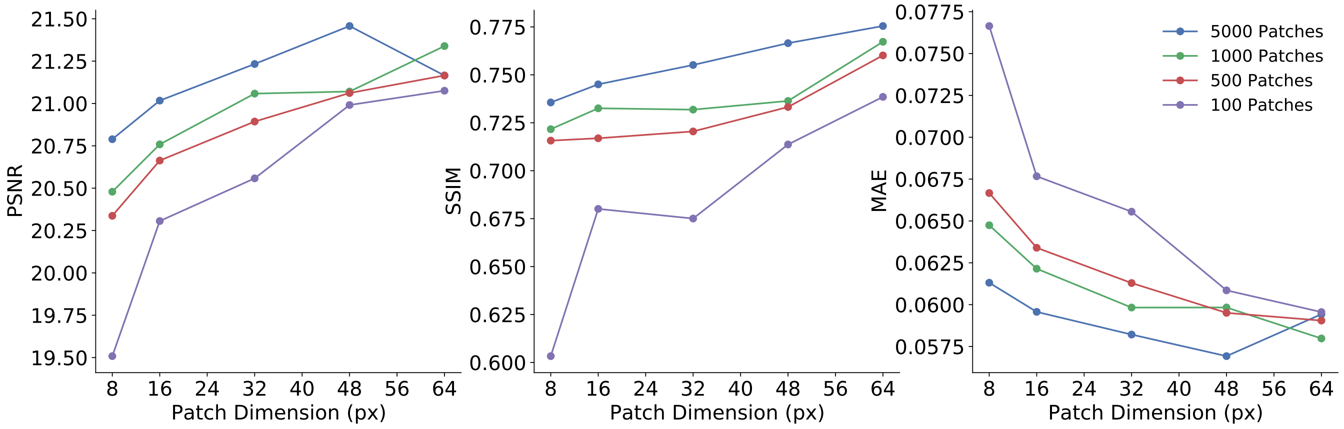

Figure S2. Performance metrics for merged scan sequences (i.e. data fusion of T1-weighted, T2-weighted, FLAIR channels) using different number of patches.

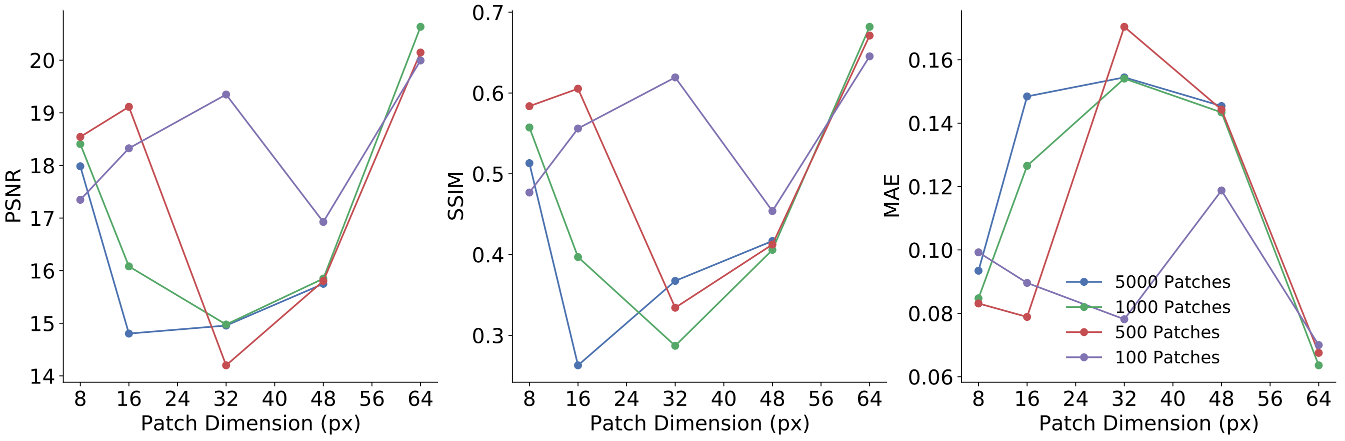

Figure S3. Performance metrics for scan sequences input separately (i.e. T1-weighted, T2-weighted, FLAIR treated independently) using different number of patches.

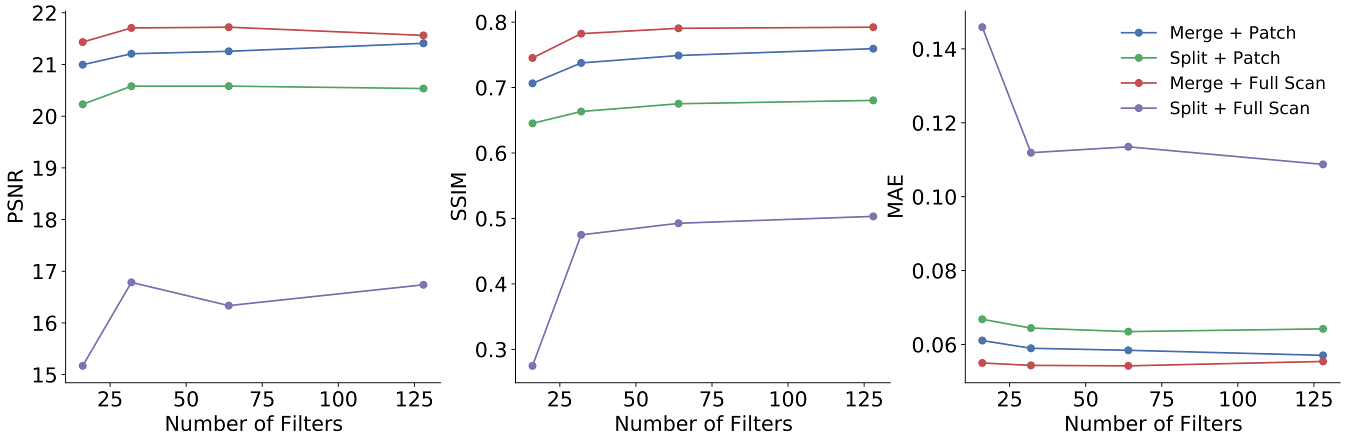

Figure S4. Performance metrics for different combinations of fused (T1-weighted, T2-weighted, and FLAIR channels) and split (T1-weighted, T2-weighted, and FLAIR treated separately) input image data entered either as full slices or in patches, in experiments that used different number of filters.

1.3 GradCAM Activation Visualisation

In Figure S5, we show the GradCAM of the critic for the best performing models, Model 0 and Model 6 for Affine and Rigid pre-processing.

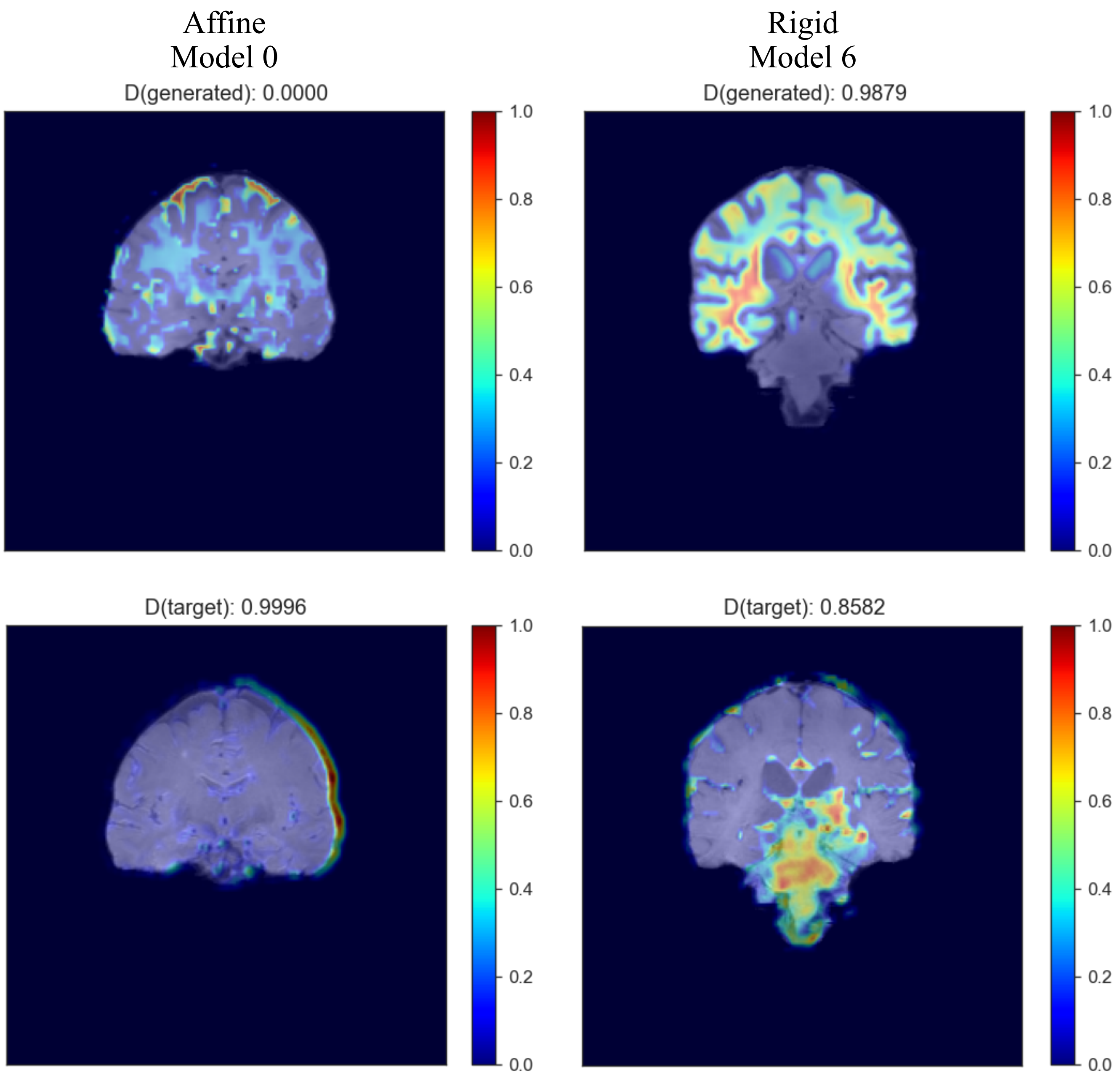

Figure S5. Example of GradCAM activation plots of the critic in (Left) Model 0 and (Right) Model 6, for images co-registered using Affine and Rigid-body registration respectively. Activation is represented in a `jet` colormap, with areas of high activation in red and low attention in blue.

1.4 Image Segmentation Results

Tables S3, S4, S5, and S6 show the volumetric results of the segmentation and the similarity metrics between the results from using each of the upsampled images against those obtained from segmenting the high resolution scan. "Upsampled affine" refers to the SR scan sequences obtained from Model 0, "upsampled rigid" refers to the SR scan sequences obtained from Model 6, "interpolated spline" refers to the scan upsampled using spline interpolation and mutual information as cost function, "interpolated sinc" refers to the scan upsampled using sinc interpolation and mutual information as cost function, and "interpolated trilinear" refers to the scan upsampled using trilinear interpolation and the correlation ratio

as cost function (default). The label of the individual scans correspond to the labels of the supplementary excel spreadsheets.

Table S3. White matter hyperintensties (WMH) volume measurements and metrics in the testing set (five patients' data) reflecting the spatial agreement between WMH binary masks segmented in upsampled and high resolution scans. Dc: Dice coefficient, TPF: true positive fraction, PPV: positive predicted value, and TNF: true negative fraction.

Group	Patient_no	Ref_vol_ml	WMH_vol_ml	WMH_Dc	WMH_TPF	WMH_PPV	WMH_TNF
Upsampled Affine	2	12.856	5.036	0.171	0.119	0.304	0.998
	9	3.231	7.939	0.216	0.373	0.152	0.996
	14	5.283	3.692	0.502	0.427	0.611	0.999
	35	7.451	4.311	0.418	0.330	0.570	0.999
	38	37.444	28.230	0.763	0.669	0.887	0.998
	58	1.899	5.757	0.045	0.091	0.030	0.997
	59	29.540	28.213	0.540	0.527	0.552	0.992
	60	2.218	10.674	0.113	0.327	0.068	0.995
	61	7.475	17.896	0.251	0.425	0.178	0.991
Upsampled Rigid	2	12.856	4.028	0.195	0.128	0.408	0.999
	9	3.231	5.309	0.178	0.235	0.143	0.997
	14	5.283	4.004	0.471	0.414	0.547	0.999
	35	7.451	2.925	0.365	0.254	0.647	0.999
	38	37.444	29.020	0.768	0.682	0.880	0.998
	58	1.899	7.383	0.051	0.124	0.032	0.996
	59	29.540	16.088	0.535	0.413	0.758	0.998
	60	2.218	7.799	0.064	0.144	0.041	0.996
	61	7.475	14.558	0.263	0.387	0.199	0.993
Interpolated Spline	2	12.856	30.130	0.232	0.388	0.166	0.986
	9	3.231	20.116	0.153	0.552	0.089	0.989
	14	5.283	13.294	0.365	0.642	0.255	0.994
	35	7.451	16.166	0.319	0.505	0.233	0.992
	38	37.444	56.467	0.643	0.806	0.535	0.983
	58	1.899	3.787	0.112	0.167	0.084	0.998
	59	29.540	35.995	0.619	0.687	0.563	0.990
	60	2.218	6.005	0.095	0.176	0.065	0.997
	61	7.475	-	-	-	-	-
Interpolated Sinc	2	12.856	29.758	0.233	0.387	0.167	0.987
	9	3.231	19.872	0.154	0.550	0.089	0.989
	14	5.283	13.147	0.366	0.639	0.257	0.995
	35	7.451	16.012	0.318	0.501	0.233	0.993
	38	37.444	56.087	0.645	0.805	0.538	0.983
	58	1.899	3.830	0.111	0.167	0.083	0.998
	59	29.540	35.668	0.620	0.685	0.567	0.990
	60	2.218	5.975	0.095	0.176	0.065	0.997
	61	7.475	-	-	-	-	-
Interpolated Trilinear	2	12.856	39.926	0.213	0.437	0.141	0.982
	9	3.231	28.574	0.123	0.606	0.069	0.983
	14	5.283	25.339	0.257	0.745	0.155	0.988
	35	7.451	22.941	0.268	0.546	0.178	0.988
	38	37.444	69.461	0.585	0.835	0.450	0.975
	58	1.899	3.613	0.117	0.170	0.089	0.998
	59	29.540	38.948	0.608	0.705	0.534	0.988
	60	2.218	7.924	0.086	0.197	0.055	0.996
	61	7.475	16.480	0.188	0.302	0.137	0.991

Table S4. Cerebrospinal fluid (CSF) volume measurements and metrics in the testing set (five patients' data) reflecting the spatial agreement between CSF binary masks segmented in upsampled and high resolution scans. Dc: Dice coefficient, TPF: true positive fraction, PPV: positive predicted value, and TNF: true negative fraction.

GROUP	PATIENT_NO	REF_VOL_ML	CSF_VOL_ML	CSF_DC	CSF_TPF	CSF_PPV	CSF_TNF
UPSAMPLED AFFINE	2	364.132	232.727	0.744	0.610	0.954	0.993
	9	215.991	175.225	0.799	0.724	0.892	0.986
	14	214.384	164.068	0.766	0.677	0.884	0.988
	35	159.727	133.431	0.742	0.681	0.815	0.983
	38	185.265	140.967	0.759	0.668	0.878	0.988
	58	118.644	108.066	0.642	0.613	0.673	0.976
	59	210.920	103.028	0.502	0.374	0.765	0.983
	60	174.790	238.210	0.738	0.872	0.640	0.951
	61	217.245	277.701	0.698	0.795	0.622	0.922
UPSAMPLED RIGID	2	364.132	187.627	0.652	0.494	0.959	0.995
	9	215.991	160.157	0.761	0.662	0.893	0.988
	14	214.384	113.870	0.622	0.476	0.897	0.993
	35	159.727	116.733	0.702	0.608	0.831	0.987
	38	185.265	139.044	0.752	0.658	0.877	0.988
	58	118.644	94.025	0.612	0.549	0.692	0.981
	59	210.920	98.705	0.485	0.356	0.761	0.983
	60	174.790	231.317	0.740	0.860	0.651	0.954
	61	217.245	277.178	0.695	0.791	0.620	0.922
INTERPOLATED SPLINE	2	364.132	320.600	0.672	0.631	0.717	0.940
	9	215.991	199.312	0.679	0.652	0.707	0.958
	14	214.384	183.446	0.576	0.535	0.623	0.956
	35	159.727	117.281	0.540	0.468	0.638	0.971
	38	185.265	193.113	0.564	0.576	0.552	0.938
	58	118.644	86.912	0.545	0.472	0.645	0.979
	59	210.920	146.631	0.595	0.504	0.726	0.971
	60	174.790	240.398	0.684	0.813	0.591	0.944
	61	217.245	-	-	-	-	-
INTERPOLATED SINC	2	364.132	319.407	0.670	0.629	0.717	0.940
	9	215.991	199.713	0.677	0.652	0.705	0.957
	14	214.384	184.945	0.576	0.536	0.622	0.955
	35	159.727	117.698	0.540	0.469	0.637	0.971
	38	185.265	193.544	0.564	0.576	0.552	0.938
	58	118.644	165.748	0.569	0.682	0.488	0.943
	59	210.920	147.327	0.595	0.505	0.724	0.971
	60	174.790	237.450	0.684	0.807	0.594	0.945
	61	217.245	161.164	0.495	0.431	0.581	0.950
INTERPOLATED TRILINEAR	2	364.132	293.370	0.692	0.625	0.776	0.956
	9	215.991	169.770	0.698	0.623	0.793	0.975
	14	214.384	164.250	0.612	0.540	0.705	0.969
	35	159.727	117.064	0.564	0.488	0.666	0.974
	38	185.265	128.168	0.602	0.509	0.736	0.976
	58	118.644	164.861	0.587	0.701	0.505	0.945
	59	210.920	297.714	0.634	0.765	0.542	0.901
	60	174.790	197.704	0.691	0.737	0.651	0.961
	61	217.245	150.216	0.580	0.491	0.710	0.968

Table S5. Venous sinuses and meningeal dura volume measurements and metrics in the testing set (five patients' data) reflecting the spatial agreement between the binary masks segmented in upsampled and high resolution scans. Dc: Dice coefficient, TPF: true positive fraction, PPV: positive predicted value, and TNF: true negative fraction.

GROUP	PATIENT_NO	REF_VOL_ML	MEN_VOL_ML	MEN_DC	MEN_TPF	EN_PPV	MEN_TNF
UPSAMPLED AFFINE	2	181.487	223.698	0.788	0.879	0.713	0.962
	9	161.764	190.394	0.783	0.852	0.724	0.963
	14	172.399	220.934	0.775	0.884	0.690	0.957
	35	179.581	192.782	0.796	0.825	0.769	0.969
	38	176.610	180.845	0.799	0.808	0.789	0.973
	58	156.837	116.055	0.735	0.640	0.864	0.989
	59	194.377	143.561	0.635	0.552	0.747	0.974
	60	176.736	160.870	0.798	0.762	0.837	0.985
	61	157.301	148.411	0.742	0.721	0.764	0.975
UPSAMPLED RIGID	2	181.487	443.607	0.529	0.911	0.323	0.835
	9	161.764	228.804	0.706	0.852	0.602	0.937
	14	172.399	350.651	0.601	0.911	0.448	0.880
	35	179.581	242.252	0.705	0.829	0.614	0.936
	38	176.610	201.023	0.761	0.814	0.715	0.959
	58	156.837	118.099	0.741	0.649	0.862	0.989
	59	194.377	158.523	0.650	0.590	0.723	0.969
	60	176.736	158.898	0.809	0.768	0.855	0.987
	61	157.301	159.777	0.757	0.763	0.751	0.972
INTERPOLATED SPLINE	2	181.487	210.247	0.705	0.761	0.657	0.957
	9	161.764	140.938	0.718	0.672	0.771	0.978
	14	172.399	152.347	0.739	0.696	0.787	0.980
	35	179.581	174.318	0.747	0.736	0.758	0.971
	38	176.610	184.908	0.668	0.683	0.653	0.954
	58	156.837	151.997	0.769	0.757	0.782	0.977
	59	194.377	124.921	0.681	0.560	0.871	0.989
	60	176.736	229.035	0.659	0.757	0.584	0.946
	61	157.301	-	-	-	-	-
INTERPOLATED SINC	2	181.487	210.272	0.704	0.760	0.656	0.957
	9	161.764	140.284	0.716	0.669	0.771	0.978
	14	172.399	151.682	0.737	0.693	0.788	0.980
	35	179.581	174.328	0.746	0.735	0.757	0.971
	38	176.610	185.344	0.666	0.682	0.650	0.954
	58	156.837	97.213	0.646	0.523	0.844	0.990
	59	194.377	124.537	0.680	0.558	0.871	0.989
	60	176.736	233.256	0.655	0.759	0.575	0.944
	61	157.301	-	-	-	-	-
INTERPOLATED TRILINEAR	2	181.487	194.894	0.611	0.634	0.590	0.953
	9	161.764	117.529	0.633	0.546	0.752	0.980
	14	219.860	127.085	0.621	0.540	0.732	0.979
	35	179.581	167.828	0.722	0.698	0.747	0.971
	38	176.610	110.496	0.594	0.483	0.772	0.982
	58	156.837	150.297	0.771	0.755	0.788	0.978
	59	194.377	122.331	0.674	0.549	0.873	0.989
	60	176.736	301.850	0.560	0.758	0.444	0.905
	61	157.301	118.263	0.650	0.569	0.757	0.980

Table S6. Pial volume measurements and metrics in the testing set (five patients' data) reflecting the spatial agreement between the binary masks segmented in upsampled and high resolution scans. Dc: Dice coefficient, TPF: true positive fraction, PPV: positive predicted value, and TNF: true negative fraction.

GROUP	PATIENT_NO	REF_VOL_ML	MEN_VOL_ML	MEN_DC	MEN_TPF	MEN_PPV	MEN_TNF
UPSAMPLED AFFINE	2	278.666	319.910	0.547	0.587	0.512	0.902
	9	175.101	219.451	0.580	0.654	0.522	0.926
	14	207.782	250.511	0.613	0.676	0.560	0.930
	35	194.529	266.621	0.583	0.691	0.504	0.908
	38	186.268	240.172	0.596	0.682	0.529	0.919
	58	145.504	175.274	0.428	0.472	0.392	0.927
	59	196.177	241.084	0.320	0.357	0.290	0.878
	60	215.893	336.770	0.429	0.548	0.352	0.873
	61	222.737	263.702	0.375	0.409	0.346	0.871
UPSAMPLED RIGID	2	278.666	249.163	0.516	0.488	0.546	0.929
	9	175.101	210.189	0.570	0.627	0.522	0.930
	14	207.782	213.674	0.570	0.578	0.562	0.941
	35	194.529	243.904	0.567	0.639	0.509	0.917
	38	186.268	233.404	0.584	0.658	0.525	0.920
	58	145.504	181.401	0.430	0.483	0.397	0.924
	59	196.177	235.795	0.313	0.344	0.286	0.880
	60	215.893	309.338	0.451	0.549	0.383	0.889
	61	222.737	247.415	0.370	0.391	0.352	0.881
INTERPOLATED SPLINE	2	278.666	321.640	0.392	0.422	0.366	0.872
	9	175.101	224.717	0.385	0.439	0.342	0.896
	14	207.782	270.986	0.379	0.437	0.335	0.885
	35	194.529	277.729	0.404	0.490	0.343	0.874
	38	186.268	216.127	0.357	0.385	0.332	0.896
	58	145.504	173.293	0.366	0.401	0.337	0.921
	59	196.177	262.106	0.360	0.420	0.314	0.871
	60	215.893	660.790	0.229	0.464	0.152	0.675
	61	222.737	-	-	-	-	-
INTERPOLATED SINC	2	278.666	319.715	0.390	0.419	0.365	0.872
	9	175.101	223.040	0.384	0.436	0.342	0.897
	14	207.782	270.065	0.379	0.436	0.335	0.886
	35	194.529	276.127	0.404	0.489	0.344	0.875
	38	186.268	214.272	0.356	0.383	0.333	0.897
	58	145.504	84.451	0.157	0.124	0.213	0.954
	59	196.177	261.503	0.359	0.419	0.314	0.872
	60	215.893	667.866	0.225	0.461	0.149	0.671
	61	222.737	116.606	0.204	0.155	0.297	0.939
INTERPOLATED TRILINEAR	2	278.666	369.649	0.389	0.453	0.341	0.847
	9	175.101	279.040	0.366	0.475	0.298	0.862
	14	207.782	342.877	0.367	0.486	0.295	0.846
	35	194.529	304.333	0.403	0.517	0.330	0.859
	38	186.268	335.198	0.398	0.557	0.310	0.834
	58	145.504	294.782	0.097	0.146	0.072	0.812
	59	196.177	39.909	0.043	0.026	0.128	0.975
	60	215.893	669.081	0.206	0.423	0.136	0.665
	61	222.737	263.411	0.390	0.426	0.360	0.875

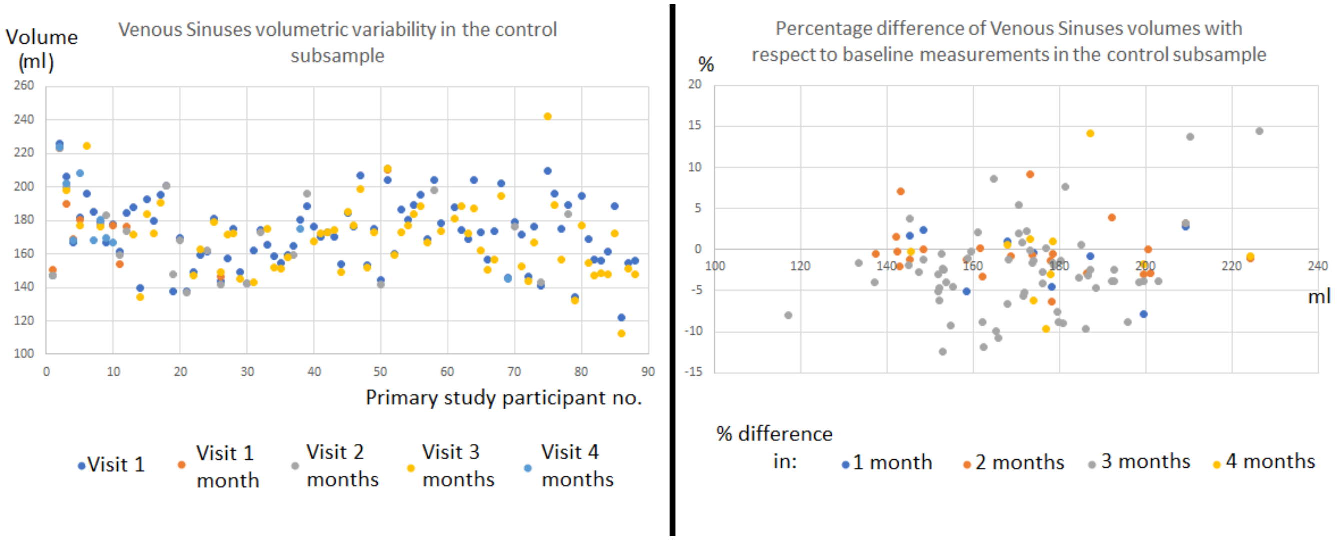

Figure S6. Volumetric variability of the venous sinuses in the control subsample throughout the 4 months after enrolling in the primary study that provided data for the present analysis.
